# Activity of nested neural circuits drives different courtship songs in *Drosophila*

**DOI:** 10.1038/s41593-024-01738-9

**Published:** 2024-08-28

**Authors:** Hiroshi M. Shiozaki, Kaiyu Wang, Joshua L. Lillvis, Min Xu, Barry J. Dickson, David L. Stern

**Affiliations:** 1 Janelia Research Campus, Howard Hughes Medical Institute, Ashburn, VA USA; 2 Queensland Brain Institute, University of Queensland, St Lucia, Queensland Australia; 3 Present Address: Lingang Laboratory, Shanghai Center for Brain Science and Brain-Inspired Intelligence Technology, Shanghai, China

**Keywords:** Neural circuits, Motor control, Sexual behaviour

## Abstract

Motor systems implement diverse motor programs to pattern behavioral sequences, yet how different motor actions are controlled on a moment-by-moment basis remains unclear. Here, we investigated the neural circuit mechanisms underlying the control of distinct courtship songs in *Drosophila*. Courting males rapidly alternate between two types of song: pulse and sine. By recording calcium signals in the ventral nerve cord in singing flies, we found that one neural population is active during both songs, whereas an expanded neural population, which includes neurons from the first population, is active during pulse song. Brain recordings showed that this nested activation pattern is present in two descending pathways required for singing. Connectomic analysis reveals that these two descending pathways provide structured input to ventral nerve cord neurons in a manner consistent with their activation patterns. These results suggest that nested premotor circuit activity, directed by distinct descending signals, enables rapid switching between motor actions.

## Abstract (web summary)

Activation of nested, but not discrete, neural circuits drives alternative courtship songs in male *Drosophila melanogaster*, providing further insight into how the nervous system can drive the same motor systems to rapidly switch between different actions.

## Main

Animals flexibly switch between different actions to adapt to a changing environment. A common mechanism to produce different actions is to activate separate neural populations, each dedicated to one action. For example, during locomotion, flexor and extensor muscles in the vertebrate limb alternately contract through activation of different neural populations in the spinal cord^1,2^. This type of motor control often underlies body movements that involve contractions of distinct populations of muscles. Diverse actions can also be produced by differentially contracting overlapping sets of muscles, as seen in the respiratory system^3^, yet little is known about how these actions are controlled by the motor system.

**Fig. 1:**
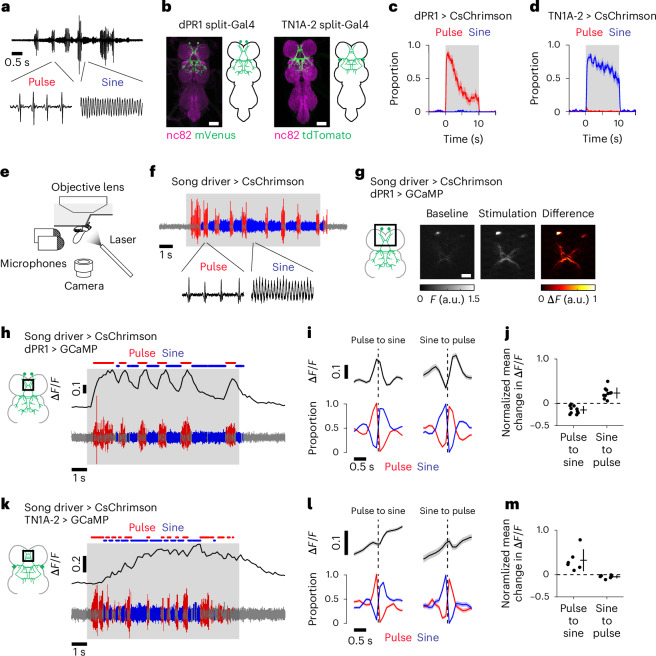
Calcium dynamics of song-promoting VNC neurons during the singing of pulse and sine song. **a**, A recording of the sound of natural *D. melanogaster* courtship song. **b**, Expression patterns of the dPR1 split-Gal4 (left) and the TN1A-2 split-Gal4 (right) in the VNC. Scale bars, 50 μm. Similar expression patterns were observed in three flies for each genotype. **c**,**d**, Results of the single fly optogenetic activation experiment. CsChrimson was expressed in either dPR1 (**c**) or TN1A-2 (**d**) neurons of male flies. **c**, Time course of the proportions of pulse (red) and sine (blue) song induced by LED stimulation of dPR1 at an irradiance of 15.9 μW mm^−2^. Data are represented as mean ± s.e.m. across flies (*n* = 12 flies). **d**, Same as **c** but for TN1A-2 (*n* = 12 flies). **e**, Schematic of calcium imaging during fly singing. **f**, Example of the sounds induced by optogenetic activation of the song driver in a fly attached to the recording plate. No thoracic dissection was made before this recording. The shaded area represents the period during which laser stimulation was applied. **g**, Example frames during recording from dPR1. Scale bar, 20 μm. **h**, Example Δ*F*/*F* trace of a part of the dPR1 neurite (top) together with the simultaneously recorded sound (bottom). **i**, Time course of dPR1 neurite Δ*F*/*F* (top) and song (bottom) around song-type transitions. Dashed vertical lines represent the timing of transitions. Data are from ten flies and represented as mean ± s.e.m. across transitions for both Δ*F*/*F* and song (*n* = 1,092 and 429 events for pulse-to-sine and sine-to-pulse transitions, respectively). **j**, The mean change in Δ*F*/*F* after song-type transitions relative to Δ*F*/*F* before the transitions (see Methods for details) for the neurite of dPR1. Each dot represents a fly. Lines represent mean ± s.d. across flies (*n* = 10 flies). *P* = 9.68 × 10^−4^ (pulse-to-sine transitions); *P* = 3.95 × 10^−4^ (sine-to-pulse transitions); two-sided one-sample *t*-test with Bonferroni correction. **k**–**m**, Same as **h**–**j** but for TN1A-2 neurite. **i**, *n* = 318 pulse-to-sine and 122 sine-to-pulse transitions in 6 flies. **j**, *n* = 6 flies. *P* = 0.048 (pulse-to-sine transitions); *P* = 0.045 (sine-to-pulse transitions). See also Extended Data Figs. 1–3.

During courtship, *Drosophila melanogaster* males vibrate their wings in specific patterns to produce acoustic communication signals called courtship song. The song is composed of two primary types, pulse and sine^4^ (Fig. 1a), which influence females’ receptivity to males in different ways^5,6^. Males dynamically switch between pulse and sine song depending on sensory feedback from females^7^ as well as based on internal states^8^. The sounds of song are shaped by wing control muscles^9^, which induce rapid changes in wing motion through coordinated firing^10^. Most of the control muscles are active during pulse song, whereas a subset of these active muscles becomes inactive during sine song^10,11^. Thus, the two song patterns are produced by recruiting overlapping, rather than distinct, sets of wing control muscles. Many possible premotor mechanisms could produce this pattern of overlapping motor activity. For example, in contrast to the motor control systems discussed above, independent neural populations can also activate overlapping muscles to produce distinct movements^12^.

Wing motor neurons extend dendrites into the ventral nerve cord (VNC)^9,11,13,14^ where they receive input from thoracic interneurons that, in turn, integrate song-promoting descending commands from the brain^4,8,15–20^. Activity of VNC interneurons is sufficient to generate song^17,21^, indicating that the circuit that generates patterned song resides in the VNC. Neurons that express the sex determination genes *fruitless* (*fru*) and/or *doublesex* (*dsx*) are involved in song production^20,22,23^, and some of these neurons appear to contribute differentially to pulse and sine song. For example, silencing the activity of dPR1 neurons reduces pulse song^20,24^, whereas silencing TN1A neurons reduces sine but not pulse song^22,24^. These findings are consistent with a model where separate populations of VNC neurons control pulse and sine song. Furthermore, a network model that incorporates these observations by positing mutually inhibitory pulse- and sine-promoting VNC neurons can generate naturalistic sequences of alternating pulse and sine songs^19^. However, it remains unclear whether such opponent activity underlies patterning of pulse and sine song because no existing preparation allows the recording of neural activity in the VNC during singing.

Here, we developed a two-photon calcium imaging assay for recording signals from neurons in the song motor circuits while flies sing alternate bouts of pulse and sine song. We found that dPR1 neurons are selectively active during pulse, whereas TN1A neurons are active during both pulse and sine song. Recordings from other song-related neurons in the VNC suggest that the nested activity patterns displayed by dPR1 and TN1A neurons are a general feature of the song circuit in the VNC. Connectomic analysis suggests that TN1A neurons can be divided into two subtypes based on their connectivity, and that dPR1 and the TN1A subtype from which we recorded calcium signals receive differential input from parallel descending pathways. Imaging the activity of these descending pathways showed that one descending neuron type, which synapses onto both dPR1 and TN1A, is active during both pulse and sine song, whereas another neuron type, which synapses primarily onto pulse-specific neurons, is selectively active during pulse. Together, our results suggest that nested neural circuits in the VNC are activated by parallel descending pathways to produce distinct acoustic communication signals in *D. melanogaster*.

## Results

### Singing involves nested activation of dPR1 and TN1A neurons

**Extended Data Fig. 1:**
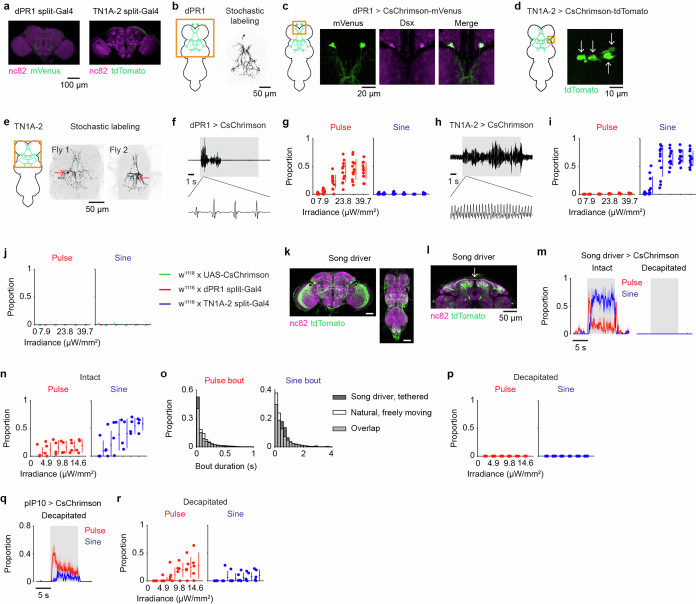
Driver lines for inducing courtship song. **a**, Expression patterns of the dPR1 split-Gal4 (left) and the TN1A-2 split-Gal4 (right) in the brain. **b**, A single neuron visualized with stochastic labeling using the dPR1 split-Gal4 line. **c**, The *dsx* gene is expressed in the neurons labeled with the dPR1 split-Gal4 line. **d**, Cell body expression pattern of CsChrimson in the TN1A-2 split-Gal4 line. Arrows indicate the location of cell bodies. **e**, Examples of single TN1A-2 neurons visualized with stochastic labeling using the TN1A-2 split-Gal4. Arrows indicate the cell bodies. **f**, Example of the sound induced by optogenetic activation of dPR1 in the single fly optogenetic activation experiment. The shaded area represents the period during which LED stimulation was applied. **g**, The mean proportions of pulse (left) and sine (right) song during the stimulation period. Each dot represents a fly. Lines represent mean ± s.d. across flies (*n* = 12 flies). **h**,**i**, Same as **f**,**g** but for TN1A-2 (*n* = 12 flies). **j**, Results for control flies in the single fly optogenetic activation experiment. The mean proportions of pulse and sine song during the stimulation period. Lines represent mean ± s.d. across flies (*n* = 11 flies for UAS-CsChrimson; *n* = 12 flies for dPR1 split-Gal4; *n* = 12 flies for TN1A-2 split-Gal4). **k**, Expression pattern of the song driver, which was used to induce pulse and sine song with optogenetic activation. Data were from a male fly. Similar expression patterns were observed in three flies. Scale bars, 50 μm. **l**, Expression pattern of the song driver at an anterior-dorsal part of the central brain of a male fly. The arrow indicates the commissural fibers of P1 neurons. **m**, Flies expressing CsChrimson under the control of the song driver were attached to the recording plate and optogenetically stimulated with a red laser. For the decapitated group, the head was removed after attachment to the recording plate. No thoracic dissection was made on these flies. The graphs show the time course of the proportions of pulse and sine song during laser stimulation at an irradiance of 12.2 μW/mm^2^. Data are represented as mean ± s.e.m. across flies (*n* = 4 flies for the intact group; *n* = 6 flies for the decapitated group). **n**, The mean proportions of pulse (left) and sine (right) song during the stimulation period for the intact group. Each dot represents a fly. Lines represent mean ± s.d. across flies (*n* = 4 flies). **o**, The distributions of the durations of pulse and sine song bouts. Dark grey bars, the songs induced by optogenetic activation with the song driver (*n* = 4 intact flies). White bars, the songs in a group of control flies in the optogenetic activation experiment shown in **j** (*w*^*1118*^ crossed to UAS-CsChrimson; *n* = 11 flies). **p**, Same as **n** but for the decapitated group (*n* = 6 flies). **q**, Flies expressing CsChrimson under the control of the split-Gal4 line for pIP10 were attached to the recording plate, decapitated, and provided with optogenetic stimulation. No dissection was made on the thorax. Graphs show time courses of the proportions of pulse and sine song during laser stimulation at an irradiance of 12.2 μW/mm^2^. Data are represented as mean ± s.e.m. across flies (*n* = 5 flies). **r**, Same as **p** but for pIP10 (*n* = 5 flies). LED, light-emitting diode.

To study the mechanisms of pulse and sine song production, we built genetic lines to label dPR1 and TN1A neurons using the split-Gal4 method^25,26^. Both dPR1 and TN1A neurons express the sex determination gene *dsx* (refs. ^20,22^). The dPR1 driver line cleanly labels a pair of VNC neurons (Fig. 1b and Extended Data Fig. 1a,b) whose cell body locations and innervation patterns match those of dPR1 (ref. ^20^). As expected, these labeled neurons express *dsx* (Extended Data Fig. 1c). The driver line for TN1A was constructed using *dsx*-DBD as a hemi-driver and thus labels a subset of *dsx*-expressing neurons. This line labels approximately four VNC neurons per hemisphere whose morphology matches that of TN1A (ref. ^22^) (Fig. 1b and Extended Data Fig. 1a,d,e). However, as we discuss in detail in a later section, using the recently published MANC connectome^27–29^ we have identified two TN1A subtypes based on connectivity: TN1A-1 and TN1A-2. The morphology of the neurons labeled by our split-Gal4 line matches the TN1A-2 subpopulation. Both dPR1 and TN1A-2 neurons also express *fru* (ref. ^24^).

dPR1 and TN1A were previously implicated in pulse and sine song production, respectively^16,20,22,24^. Indeed, when we expressed the light-gated cation channel CsChrimson^30^ in dPR1 neurons of solitary males, optogenetic activation acutely elicited pulse but not sine song over a range of stimulation intensities (Fig. 1c and Extended Data Fig. 1f,g,j). On the other hand, activation of TN1A-2 induced sine but not pulse song^24^ (Fig. 1d and Extended Data Fig. 1h–j). Thus, dPR1 and TN1A-2 are capable of inducing pulse and sine song, respectively.

**Extended Data Fig. 2:**
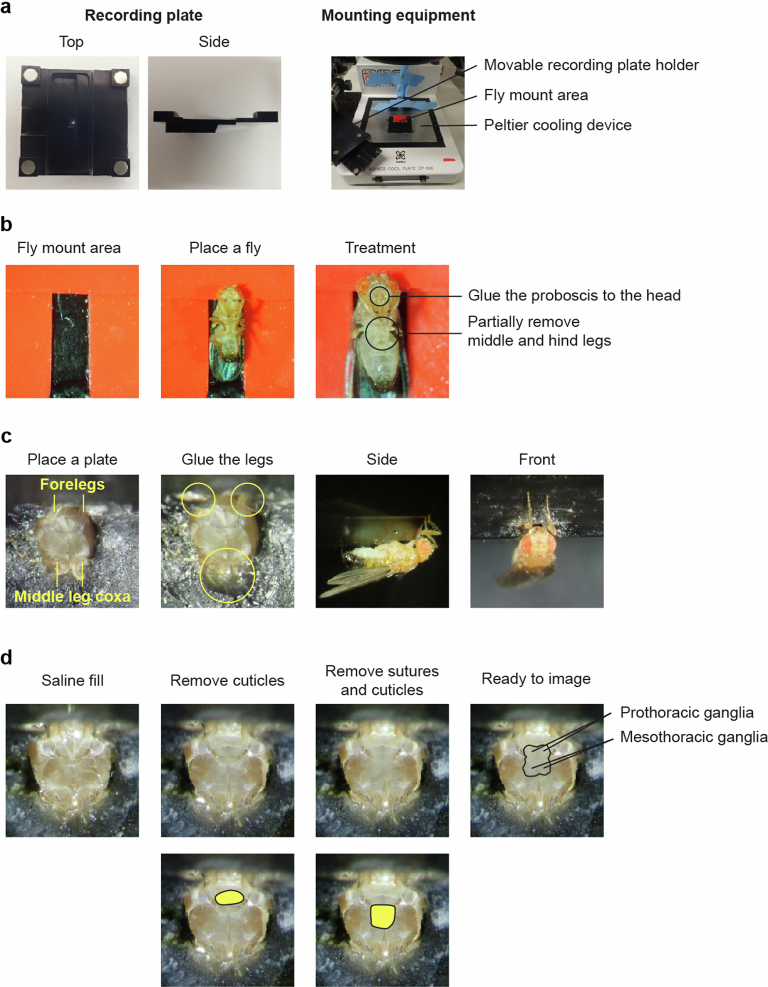
Preparation for calcium imaging from the VNC in singing flies. **a**, Equipment. **b**, A cold-anesthetized fly was placed on a Peltier plate held at 4 °C. Middle and hind legs were cut with forceps around the femur-trochanter junctions. The proboscis was glued to the head capsule. **c**, Using a manual manipulator attached to the plate holder, the recording plate was positioned above the fly so that the part of the thorax between the forelegs and middle legs is visible through the central hole of the plate. The fly was attached to the plate by applying glue to the coxa and trochanter of the middle legs and femur of the forelegs. The fly was held at room temperature afterward. **d**, For dissection, the top side of the plate was filled with saline. The cuticle and the sutures covering the pro- and meso-thoracic ganglia of the VNC were removed with fine forceps. Care must be taken not to deform the thorax during dissection, which could significantly reduce singing behavior during calcium imaging.

We next examined the activity of dPR1 and TN1A-2 neurons during pulse and sine song. Before this study, there had been no preparation that allowed recording the activity of VNC neurons while flies sang. This partly stems from two technical challenges. First, wing vibration is dependent on muscle-induced oscillatory movements of the thorax^31^ and thus attaching the thorax, which houses the VNC, to the recording apparatus often disturbs these oscillations. Second, wing muscles are adjacent to the VNC and are easily damaged during the dissection that is required to image the VNC. To overcome these challenges, we glued parts of the legs, instead of the thorax, to the recording plate and dissected the ventral side of the thorax where wing muscles are absent (Fig. 1e and Extended Data Fig. 2). This assay provides optical access to VNC neurons while allowing approximately normal wing vibrations for song production.

Song was induced by optogenetic activation of neurons labeled by a LexA line (R22D03-LexA) that we term the song driver (Extended Data Fig. 1k). This driver line labels multiple neurons including the male-specific brain neurons P1 (Extended Data Fig. 1l), which integrate multisensory cues and promote the initiation and persistence of courtship behavior^15,18,20,32–36^. Optogenetic activation of the neurons labeled by the song driver in tethered males induced alternating bouts of pulse and sine song as observed in natural singing, even though the activation light was maintained at constant power (Fig. 1f and Extended Data Fig. 1m,n). The bout durations of the induced pulse and sine song were similar to those produced during normal courtship in freely moving flies (Extended Data Fig. 1o). Removal of the head eliminated the production of song during optogenetic stimulation (Extended Data Fig. 1m,p). This effect was not caused by decapitation-induced physical damage because optogenetic activation of pIP10—a song-promoting descending neuron^4,8,15–20,24^ —in the same preparation elicited song in decapitated flies (Extended Data Fig. 1q,r). These results indicate that neurons labeled by this driver line in the VNC are not sufficient to drive song and that neurons labeled by this line in the brain (which includes P1 neurons) are required to induce song by optogenetic stimulation.

**Extended Data Fig. 3:**
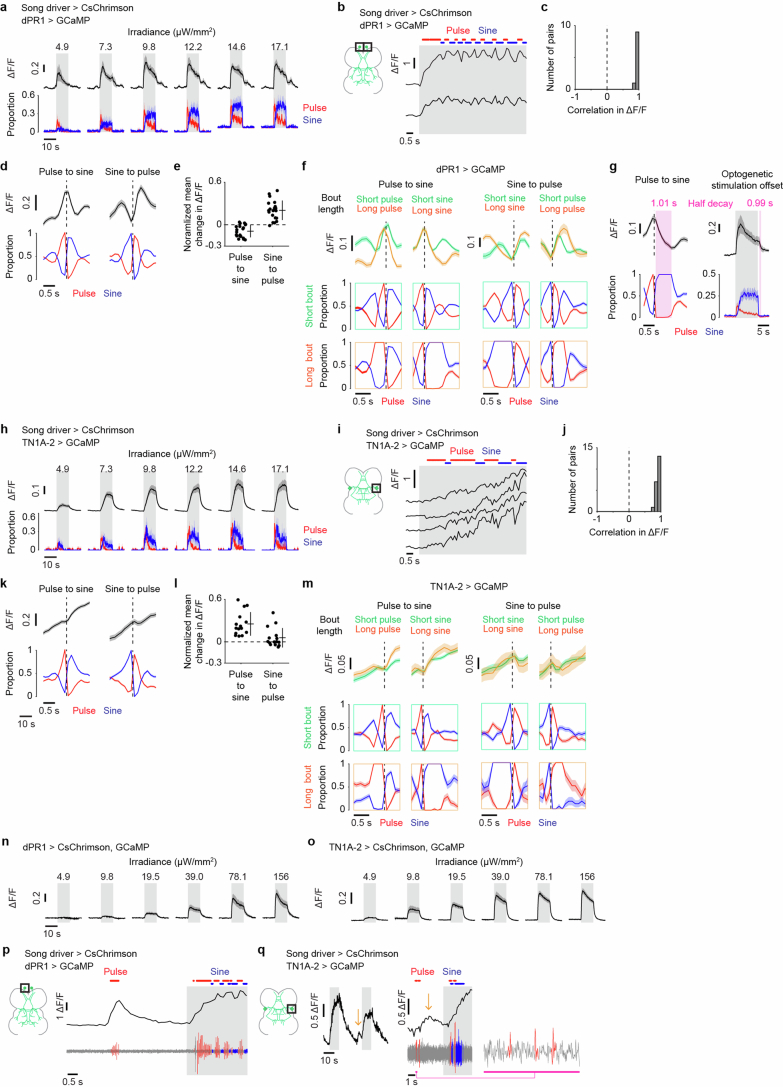
Calcium dynamics of song-promoting VNC neurons during the singing of pulse and sine song. **a**, Time course of ΔF/F recorded from dPR1 neurite (top) and the proportions of pulse and sine song (bottom) during the optogenetic activation of the song driver. The shaded area represents the period during which the optogenetic stimulation was applied. The mean ΔF/F trace and the song proportions were calculated for each fly and then averaged across flies. Data are represented as mean ± s.e.m. across flies (*n* = 10 flies). **b**, Example ΔF/F traces of two dPR1 neurons recorded simultaneously. Calcium signals at cell bodies were analyzed to extract single neuron signals. The song driver was used to express CsChrimson. The shaded area represents the period during which laser stimulation was applied. **c**, Histogram of the Pearson’s correlation coefficients between pairs of dPR1 neurons recorded simultaneously (*n* = 10 pairs in 10 flies). **d**, Time course of dPR1 somatic ΔF/F and song around song-type transitions. Dashed vertical lines represent the transition times. Data are from 20 neurons in 10 flies and represented as mean ± s.e.m. across transitions for both ΔF/F and song (n = 2,184 and 858 events for pulse-to-sine and sine-to-pulse transitions, respectively). **e**, The mean change in ΔF/F after song-type transitions relative to ΔF/F before the transitions for dPR1. Each dot represents a neuron. Lines represent mean ± s.d. across neurons (*n* = 20 neurons in 10 flies). *P* = 5.09 × 10^−4^ (pulse-to-sine transitions); *P* = 1.81 × 10^−6^ (sine-to-pulse transitions); two-sided one-sample t test with Bonferroni correction. **f**, Time course of dPR1 neurite ΔF/F (top) and song (bottom) around song-type transitions plotted separately for short and long bout lengths, where bouts longer than 422.3 ms (3 bins) and 703.8 ms (5 bins) were defined as long pulse and sine bouts, respectively. Dashed vertical lines represent the timing of transitions. Data are from 10 flies and represented as mean ± s.e.m. across transitions for both ΔF/F and song (*n* = 98–884 events for each curve). **g**, Half decay time of the dPR1 neurite GCaMP signals during pulse-to-sine transitions (left) and after the offset of the song driver stimulation (right). Pink shaded areas represent the periods from which the decay times were calculated. Left, Time course of dPR1 neurite ΔF/F (top) and song (bottom) around pulse-to-sine transitions of long sine bouts. The same data as in **f**. Right, Time course of dPR1 neurite ΔF/F (top) and the proportions of pulse and sine song (bottom) during optogenetic activation of the song driver. The mean ΔF/F trace and the song proportions were calculated for each fly by averaging trials of all the stimulation strengths, followed by averaging across flies. Data are represented as mean ± s.e.m. across flies (*n* = 10 files). **h–m**, Same as **a–f** but for TN1A-2 neurons. (**h**) *n* = 6 flies. (**i**) Example of ΔF/F traces of four TN1A-2 neurons recorded simultaneously. (**j**), *n* = 21 pairs in 4 flies. (**k**), *n* = 967 (pulse-to-sine) and 444 (sine-to-pulse) transitions from 15 neurons in 4 flies. (**l**) *n* = 15 neurons in 4 flies. *P* = 7.79 × 10^−5^ (pulse-to-sine transitions); *P* = 0.25 (sine-to-pulse transitions). (**m**) Data are from 6 flies (*n* = 18–281 events for each curve). **n**, CsChrimson and jGCaMP7f were expressed in the same neurons and calcium responses to optogenetic stimulation were recorded. Shown is the time course of ΔF/F recorded from dPR1 neurite. The same format as in **a** (*n* = 6 flies). **o**, Same as f but for TN1A-2 neurites (*n* = 6 flies). **p**, Example ΔF/F trace of a single dPR1 neuron (top) together with the simultaneously recorded sound (bottom). The fly produced a train of pulse song outside the stimulation period, which was accompanied by an increase in calcium signals. **q**, Example ΔF/F trace of a single TN1A-2 neuron. The fly produced pulse song outside the stimulation period, which was followed by an increase in calcium signals (arrows).

To characterize the activity of dPR1 and TN1A-2 during pulse and sine song, we performed two-photon imaging of the genetically encoded calcium indicator jGCaMP7f (ref. ^37^) expressed in dPR1 or TN1A-2 neurons while inducing song by optogenetic stimulation of the song driver. Optogenetic stimulation acutely increased calcium signals of dPR1 neurons in both hemispheres (Fig. 1g,h, Extended Data Fig. 3a–c and Supplementary Video 1). Larger calcium signals were accompanied by more song, consistent with a role of dPR1 in song production (Extended Data Fig. 3a). Notably, over the course of constant optogenetic stimulation, the dPR1 calcium signals repeatedly increased and decreased as the fly alternated between song types (Fig. 1h and Extended Data Fig. 3b). The calcium signal increased during transitions from sine to pulse and decreased during transitions from pulse to sine (Fig. 1h–j and Extended Data Fig. 3d,e). Longer pulse and sine bouts were associated with greater increases and decreases, respectively, in calcium signals (Extended Data Fig. 3f), indicating that dPR1 activity reflects the song type being sung rather than song-type switch events. The decay rate of the calcium signal during sine song was similar to the decay rate observed immediately after the offset of optogenetic stimulation, when flies stopped singing (Extended Data Fig. 3g), suggesting that dPR1 neurons are not active during sine song. The preference of dPR1 for pulse song is consistent with the observation that optogenetic activation of dPR1 promotes pulse song (Fig. 1c).

Similar to the observations we made of dPR1, calcium signals of TN1A-2 neurons increased in response to optogenetic stimulation of the song driver (Fig. 1k, Extended Data Fig. 3h,i and Supplementary Video 2). Multiple TN1A-2 neurons that were recorded simultaneously showed highly correlated calcium signals (Extended Data Fig. 3j), suggesting that these neurons encode similar information. Consistent with the observation that optogenetic activation of TN1A-2 promotes sine song (Fig. 1d), TN1A-2 calcium signals increased when the song type switched from pulse to sine (Fig. 1k–m and Extended Data Fig. 3k–m). However, TN1A-2 calcium signals decreased only slightly when the song type switched from sine to pulse (Fig. 1k–m and Extended Data Fig. 3k–m). This pattern contrasts with the dPR1 calcium signals, which rapidly decreased after the transition to the nonpreferred song type (Fig. 1h,i). These results suggest that TN1A-2 neurons are active during both pulse and sine song with a preference for sine.

Optogenetic activation of the song driver induced pulse song most frequently right after the stimulation onset, whereas the proportion of sine song increased over time (Extended Data Fig. 3a,h). Consistent with their song preferences, the calcium signal in dPR1 peaked soon after the stimulation onset while the TN1A-2 signal developed over several seconds (Extended Data Fig. 3a,h). When these neurons were directly activated with optogenetics, the time course of calcium signals in both cell types was similar to that of dPR1 during song driver stimulation (Extended Data Fig. 3n,o). This suggests that time-varying input to TN1A-2 neurons contributes to more abundant sine song after prolonged stimulation.

The tethered flies occasionally produced pulse song outside of the optogenetic stimulation periods, as observed during intermittent stimulation of P1 neurons in freely moving isolated male flies^15,18^, which allowed us to determine whether the activity patterns we had observed for dPR1 and TN1A-2 during pulse song were dependent on activation of the song driver. We found that both dPR1 and TN1A-2 showed increased calcium signals when flies sang pulse song outside of the optogenetic stimulation periods (Extended Data Fig. 3p,q). This result further supports the idea that pulse song involves activation of both dPR1 and TN1A-2.

**Fig. 2:**
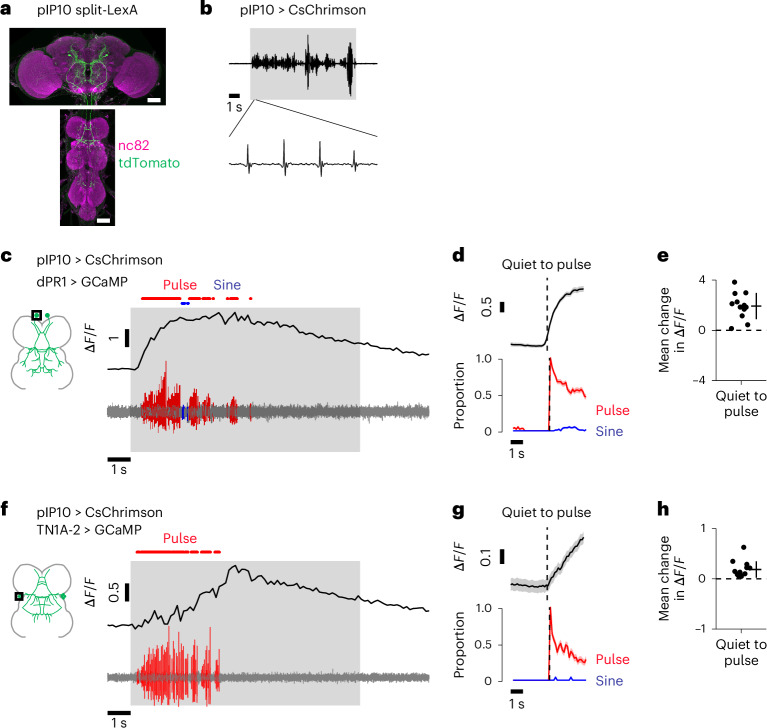
dPR1 and TN1A-2 are activated during pulse song induced by stimulation of the descending neurons pIP10. **a**, Expression pattern of the split-LexA driver for pIP10. Similar expression patterns were observed in three flies. Scale bars, 50 μm. **b**, Example of the sound induced by optogenetic activation of pIP10 in the single fly optogenetic activation experiment. The shaded area represents the period of LED stimulation. **c**, Example Δ*F*/*F* trace of a dPR1 neuron (top) together with the simultaneously recorded sound (bottom). jGCaMP7s was expressed with *dsx*-Gal4 and calcium signals were recorded from a dPR1 cell body. **d**, Time course of dPR1 Δ*F*/*F* and song around transitions from quiet to pulse song production. Dashed vertical lines represent the transition time. Data are from 12 neurons in 6 flies and represented as mean ± s.e.m. across transitions for both Δ*F*/*F* and song (*n* = 190 transitions). **e**, The mean change in Δ*F*/*F* after quiet-to-pulse transitions (see Methods for details) for dPR1. Each dot represents a neuron. Lines represent mean ± s.d. across neurons (*n* = 12 neurons in 6 flies). **f–h**, Same as **c–e** but for TN1A-2. GCaMP was expressed with the TN1A-2 split-Gal4 line. **g**, *n* = 99 transitions from 13 neurons in 4 flies. **h**, *n* = 13 neurons in 4 flies. See also Extended Data Fig. 4.

**Extended Data Fig. 4:**
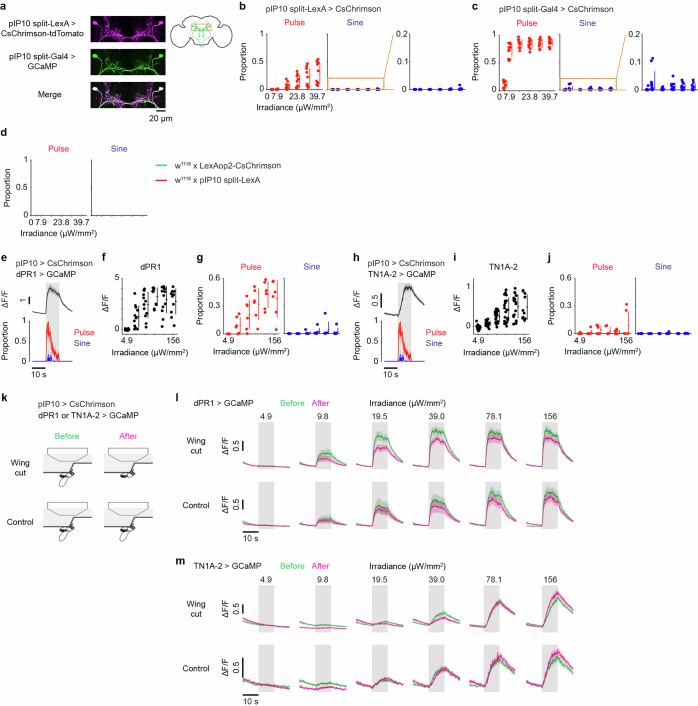
dPR1 and TN1A-2 are activated during pulse song induced by stimulation of the descending neurons pIP10. **a**, Expression patterns of the pIP10 split-LexA and split-Gal4 drivers around the cell bodies of pIP10. Similar expression patterns were observed in three flies. **b**, The mean proportions of pulse and sine song during the stimulation period in the single fly optogenetic activation experiment with the pIP10 split-LexA line. Each dot represents a fly. Lines represent mean ± s.d. across flies (*n* = 12 flies). **c**, Same as **b** but for the pIP10 split-Gal4 line (*n* = 12 flies). **d**, Results for control flies in the single fly optogenetic activation experiment. The mean proportions of pulse and sine song during the stimulation period. Lines represent mean ± s.d. across flies (*n* = 12 flies for each genotype). **e**, Time course of ΔF/F recorded from dPR1 and the proportions of song for the laser stimulation at the irradiance of 156.2 μW/mm^2^. jGCaMP7s was expressed with *dsx*-Gal4 and calcium signals were recorded from dPR1 cell bodies. Data are represented as mean ± s.e.m. across neurons (top; *n* = 12 neurons in 6 flies) and flies (bottom; *n* = 6 files). **f**, The mean ΔF/F of dPR1 during the stimulation of pIP10. Each dot represents a neuron. Lines represent mean ± s.d. across neurons (*n* = 12 neurons in 6 flies). The X-axis is on a logarithmic scale. **g**, The mean proportions of pulse and sine song during the stimulation period in the recordings from dPR1. Each dot represents a fly. Lines represent mean ± s.d. across flies (*n* = 6 flies). **h–j**, Same as **e–g** but for TN1A-2. (**h,i**) *n* = 19 neurons in 6 flies. (**j**) *n* = 6 flies. **k**, Schematic of the wing cut experiment. **l**, Time course of ΔF/F recorded from dPR1 cell bodies for the wing cut (top) and the control (bottom) groups. The shaded area represents the period of the optogenetic stimulation. The mean ΔF/F trace for each irradiance was calculated for each fly and then averaged across flies. Data are represented as mean ± s.e.m. across flies (*n* = 6 flies for each group). **m**, Same as **l** but for TN1A-2 (n = 6 flies for each group).

As an independent test of pulse-related activity, we recorded calcium signals from dPR1 and TN1A-2 while pulse song was induced by activating pIP10, a pair of song-promoting descending neurons^4,8,15–20^. We expressed CsChrimson in pIP10 using a new split-LexA line that cleanly labels pIP10 (Fig. 2a and Extended Data Fig. 4a). In freely moving solitary males, pIP10 activation with the split-LexA line induced some pulse song and almost no sine song (Fig. 2b and Extended Data Fig. 4b,d), whereas activation with the pIP10 split-Gal4 line drove high levels of pulse song and some sine song^17,24^ (Extended Data Fig. 4c,d). This is likely due to higher levels of CsChrimson expression in the split-Gal4 line. To examine neural activity during pulse song, we optogenetically activated pIP10 neurons with the split-LexA line while recording jGCaMP7s signals in dPR1 or TN1A-2. Both dPR1 and TN1A-2 showed increased calcium signals when flies sang pulse song (Fig. 2c–h and Extended Data Fig. 4e–j). Wing removal had little impact on the increased calcium signals during optogenetic stimulation (Extended Data Fig. 4k–m), indicating that sensory feedback from the wings is not the dominant source of the signals. Taken together, our data suggest that singing multiple songs involves nested activation of dPR1 and TN1A-2, since both cell types are active during pulse song while only TN1A-2 neurons are active during sine song.

### TN1 neurons exhibit nested activation during singing

**Fig. 3:**
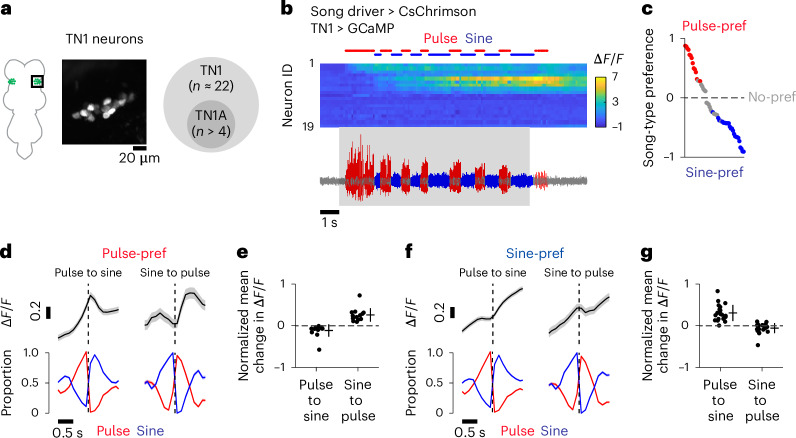
Calcium dynamics of TN1 neurons during the singing of pulse and sine song. **a**, Left, example calcium signals from the cell bodies of TN1 neurons in one hemisphere in vivo. GCaMP was driven by *dsx*-Gal4. The image is a maximum projection of a stack of ten *z*-planes. Right, there are approximately 22 TN1 neurons in each hemisphere of the VNC^38^. TN1A neurons are a subset of TN1 neurons. **b**, Example Δ*F*/*F* of a population of TN1 cell bodies recorded simultaneously in a single individual (top) together with the sound recording (bottom). The song driver was used to express CsChrimson. The shaded area represents the period where laser stimulation was applied. **c**, The distribution of the song-type preference (*n* = 56 neurons in 4 flies), which quantifies how much calcium signals differed between pulse and sine song around song-type transitions (see Methods for details). Red and blue dots indicate neurons whose calcium signals were significantly higher for pulse and sine, respectively (*P* < 0.05, two-sided permutation test). **d**, Time course of Δ*F*/*F* of pulse-preferring TN1 neurons (top) and song (bottom) around song-type transitions. Dashed vertical lines represent the transition time. Data are from 13 neurons in 3 flies and represented as mean ± s.e.m. across transitions for both Δ*F*/*F* and song (*n* = 825 and 315 events for pulse-to-sine and sine-to-pulse transitions, respectively). **e**, The mean change in Δ*F*/*F* after song-type transitions relative to Δ*F*/*F* before the transitions (see Methods for details) for pulse-preferring TN1 neurons. Each dot represents a neuron. Lines represent mean ± s.d. across neurons (*n* = 13 neurons in 3 flies). *P* = 0.034 (pulse-to-sine transitions); *P* = 1.82 × 10^−4^ (sine-to-pulse transitions); two-sided one-sample *t*-test with Bonferroni correction. **f**,**g**, Same as **d** (**f**) and **e** (**g**) but for sine-preferring TN1 neurons. **f**, *n* = 1,370 (pulse-to-sine) and 511 (sine-to-pulse) transitions from 25 neurons in 4 flies. **g**, *n* = 21 neurons in 4 flies. Four neurons were excluded from this analysis because the mean Δ*F*/*F* before song-type transitions was too low (<0.1) to reliably estimate the normalized change in Δ*F*/*F*. *P* = 1.02 × 10^−6^ (pulse-to-sine transitions); *P* = 0.089 (sine-to-pulse transitions). See also Extended Data Fig. 5. Pref, preference.

To examine whether the combinatorial activation pattern observed in dPR1 and TN1A-2 is a general property of pulse- and sine-preferring neurons in the VNC, we focused on TN1 neurons. These neurons are male-specific, *dsx*-expressing VNC neurons involved in song production^19,22,38^. There are approximately 22 TN1 neurons in each hemisphere^38^, which are composed of at least five anatomically defined cell types including TN1A (ref. ^22^) (Fig. 3a). While manipulation of the activity of TN1 neurons primarily influences sine song, a subset of TN1 neurons influences pulse song^19,22,24^.

**Extended Data Fig. 5:**
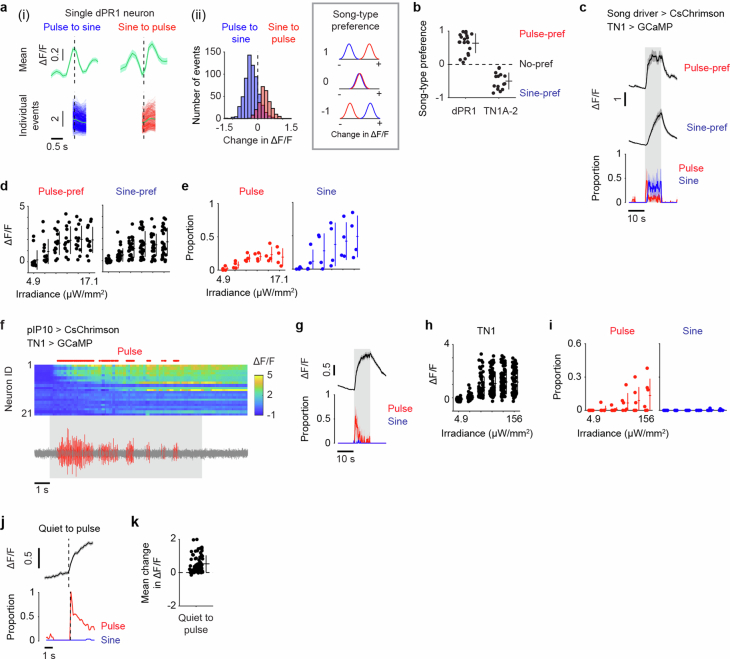
Calcium dynamics of TN1 neurons during the singing of pulse and sine song. **a**, Calculation of the song-type preference for an example dPR1 neuron. This index quantifies the selectivity for pulse and sine song during song-type transitions for individual neurons and is calculated in two steps (see Methods for detail). (i) First, the time course of ΔF/F around the transition is calculated for pulse-to-sine and sine-to-pulse transitions. As an example, the mean ΔF/F across events (top; the shaded areas represent s.e.m. across events, *n* = 640 pulse-to-sine transitions and 276 sine-to-pulse transitions) and individual ΔF/F traces for each event (bottom) around song-type transitions are shown for one dPR1 neuron. Dashed vertical lines represent the transition time. For individual traces, only the part of the data used in the calculation of the index is shown. (ii) Second, histograms are calculated for the change in ΔF/F around the song-type transitions for pulse-to-sine (blue) and sine-to-pulse (red) transitions and the song-type preference is calculated using the receiver operating characteristics analysis. The song-type preference for this example dPR1 neuron was 0.87. **b**, Histograms for the song-type preference for dPR1 (*n* = 20 neurons in 10 flies) and TN1A-2 (*n* = 15 neurons in 4 flies) calculated using the data shown in Extended Data Fig. 3d, e, k, l. Lines represent mean ± s.d. across neurons. **c**, Time course of ΔF/F recorded from pulse-preferring and sine-preferring TN1 neurons (top) and the proportions of pulse and sine song (bottom) for the laser stimulation at an irradiance of 9.8 μW/mm^2^. Data are represented as mean ± s.e.m. across neurons (top; *n* = 13 pulse-preferring in 3 flies and 25 sine-preferring neurons in 4 flies) and flies (bottom; *n* = 4 files). **d**, The mean ΔF/F for pulse-preferring and sine-preferring TN1 neurons during the stimulation of the neurons labeled by the song driver. Each dot represents a neuron. Lines represent mean ± s.d. across neurons (*n* = 13 pulse-preferring and 25 sine-preferring neurons in 4 flies for each irradiance). **e**, The mean proportions of pulse and sine song during the stimulation period for the data shown in **d** (*n* = 4 flies for each irradiance). Each dot represents a fly. **f–k**, Calcium imaging from TN1 cell bodies during simulation of pIP10. CsChrimson was expressed using the pIP10 split-LexA driver. (**f**) Example ΔF/F of a population of TN1 neurons recorded simultaneously (top) together with the sound recording (bottom). The shaded area represents the period where laser stimulation was applied. (**g**) Top, Time course of ΔF/F for the TN1 neurons that showed significant responses to optogenetic stimulation (*P* < 0.05, two-sided *t*-test; *n* = 77 neurons in 4 flies) for the laser stimulation at the irradiance of 78.1 μW/mm^2^. Bottom, The proportions of song during the recording from these neurons. Data are represented as mean ± s.e.m. across neurons (top) and flies (bottom). (**h**) The mean ΔF/F for pulse-preferring and sine-preferring TN1 neurons during stimulation of pIP10. Each dot represents a neuron. Lines represent mean ± s.d. across neurons (*n* = 77 neurons in 4 flies). The X-axis is in a logarithmic scale. (**i**) The mean proportions of pulse and sine song during the stimulation period for the data shown in **h** (*n* = 4 flies). Each dot represents a fly. (**j**) Time course of TN1 ΔF/F and song around transitions from quiet to pulse song production. Dashed vertical lines represent the transition times. Data are from 77 neurons in 4 flies and represented as mean ± s.e.m. across transitions for both ΔF/F and song (*n* = 869 transitions). (**k**) The mean change in ΔF/F after quiet-to-pulse transitions (see Methods for detail) for TN1. Each dot represents a neuron. Lines represent mean ± s.d. across neurons (*n* = 77 neurons in 4 flies).

To characterize the activity of TN1 neurons during song, we recorded calcium signals from a large fraction of individual TN1 neurons in each fly using the *dsx*-Gal4 line^39^ (Fig. 3a,b) while inducing song by optogenetic stimulation of the song driver. The optogenetic stimulation elicited significant changes in calcium signals (*P* < 0.05, *t*-test; see Methods for details) in 69.1% of the recorded TN1 neurons (56 of 89 neurons in 4 flies). For each neuron with significant responses, we quantified the selectivity for song type with an index we termed the song-type preference (Fig. 3c and Extended Data Fig. 5a,b). Most TN1 neurons showed significant preference for pulse or sine song (38 of 56 neurons in 4 flies; *P* < 0.05, permutation test), with most preferring sine song (*n* = 25 of 38 neurons in 4 flies) (Fig. 3c and Extended Data Fig. 5c–e). The average number of sine-preferring neurons in each fly (*n* = 6.3 neurons per hemisphere) was larger than the number of neurons labeled by the TN1A-2 split-Gal4 driver (*n* = 4 neurons per hemisphere), suggesting that the sine-preferring population includes neurons that were not labeled with the TN1A-2 split-Gal4 line discussed earlier.

As observed for dPR1 and TN1A-2, pulse- and sine-preferring TN1 neurons displayed increased calcium signals during optogenetic stimulation of the song driver (Fig. 3b and Extended Data Fig. 5c–e). To examine the pulse- and sine-related activity, we calculated the average calcium signals during song-type transitions for each population. We found that both populations showed increased calcium signals when the song switched to the preferred type (Fig. 3d–g). However, pulse- but not sine-preferring neurons showed decreased calcium signals when the song switched to the nonpreferred type (Fig. 3d–g). This result suggests that—as we observed for dPR1 and TN1A-2 neurons—both pulse- and sine-preferring TN1 neurons are active during pulse song, while only the sine-preferring neurons show high activity levels during sine song.

To further characterize pulse-related activity in TN1 neurons, we drove pulse song by optogenetically activating pIP10, which primarily drives pulse song, while recording calcium signals from TN1 neurons. Optogenetic stimulation of pIP10 induced changes in calcium signals in a large fraction of TN1 neurons (77 of 83 neurons in 4 flies; *P* < 0.05, *t*-test; see Methods for details), of which virtually all neurons displayed increased calcium signals when the flies produced pulse song (76 of 77 neurons; Extended Data Fig. 3f–k). This result further supports the idea that pulse song involves the coactivation of pulse-preferring and sine-preferring TN1 neurons during pulse song.

### Nested activation is found throughout the song motor circuit

**Fig. 4:**
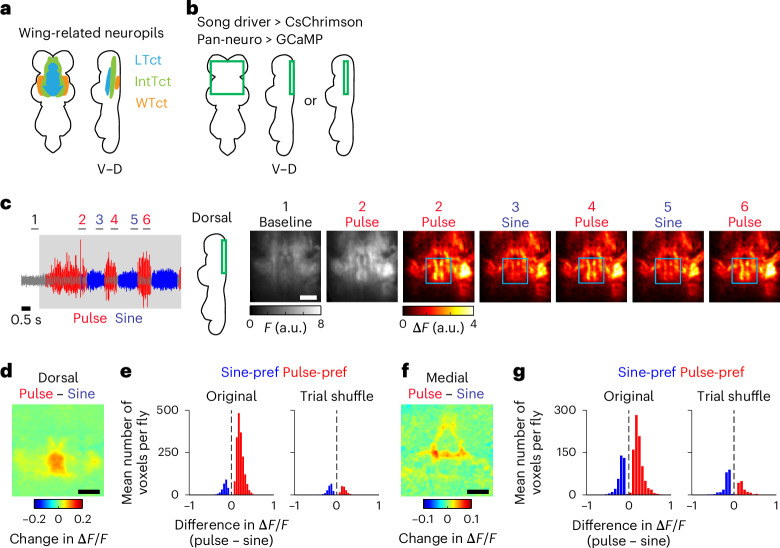
Pan-neuronal calcium dynamics in the song motor circuit during the singing of pulse and sine song. **a**, Schematic of the wing-related neuropils. **b**, Schematic of the region imaged. For each fly, images (10 *z*-planes at a 4-μm step) were obtained from either a dorsal or medial part of the VNC. CsChrimson and GCaMP were expressed using the song and pan-neuronal drivers, respectively. **c**, Left, the sound data from an example recording. Bars at the top indicate periods during which example calcium imaging frames, shown on the right, are presented. The shaded area represents the period during which laser stimulation was applied. Right, example frames. For each period, images were averaged across *z*-planes and time. Blue boxes highlight the region that showed strong song-selective signals. Scale bar, 50 μm. **d**, Population-averaged Δ*F*/*F* for the difference between pulse and sine song around song-type transitions for the recordings from the dorsal part of the VNC (*n* = 9 flies). Scale bar, 50 μm. **e**, Histogram of the mean difference in Δ*F*/*F* between pulse and sine song during song-type transitions for the voxels that changed Δ*F*/*F* depending on song type (*n* = 9 flies; *P* < 0.001, two-sided permutation test). To obtain a histogram expected by chance, the same analysis was conducted after randomizing the relationship between song and calcium signals (‘Trial shuffle’; Methods). **f**,**g**, Same as **d** (**f**) and **e** (**g**) but for the recordings from the medial part of the VNC (*n* = 3 flies). See also Extended Data Fig. 6. LTct, lower tectulum; IntTct, intermediate tectulum; WTct, wing tectulum; D, dorsal; V, ventral.

**Extended Data Fig. 6:**
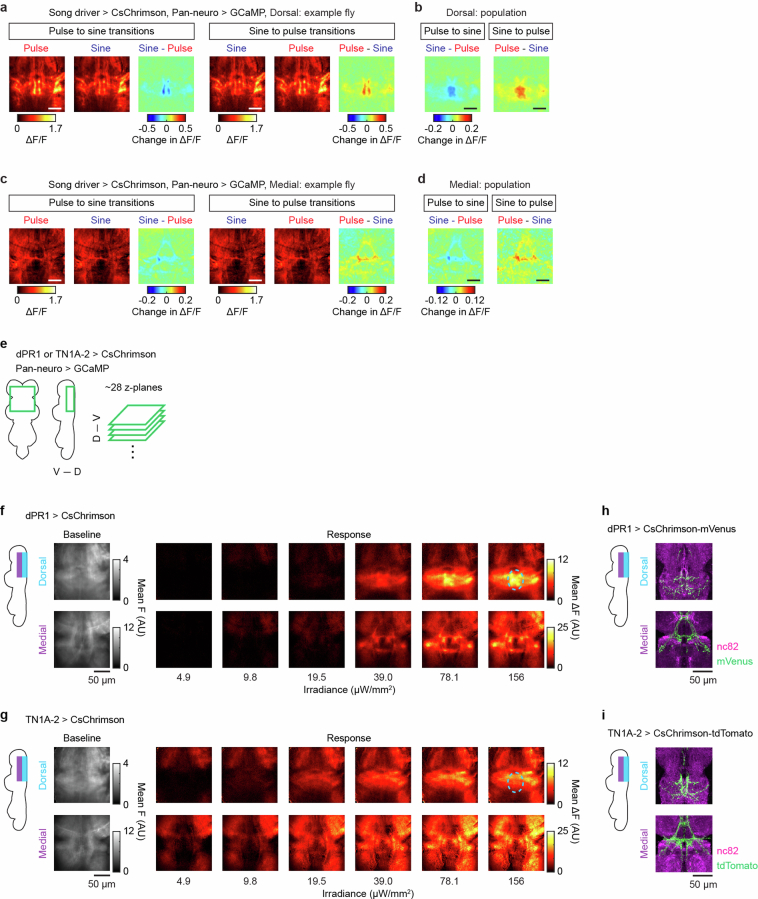
Pan-neuronal calcium dynamics in the song motor circuit during the singing of pulse and sine song. **a**, Changes in calcium signals around song-type transitions for an example fly. Calcium signals were recorded from a dorsal part of the VNC. Left, average frames during pulse to sine transitions (*n* = 71 transitions). Images were averaged across z-planes and time. Right, average frames during sine to pulse transitions (*n* = 29 transitions). Scale bars, 50 μm. **b**, Population averaged changes in ΔF/F during song-type transitions for the recordings from the dorsal part of the VNC (*n* = 9 flies). Scale bars, 50 μm. **c,d**, Same as **a,b** but for the recordings from the medial part (*n* = 3 flies). (c) n = 171 and 62 pulse-to-sine and sine-to-pulse transitions, respectively. **e**, Schematic of the VNC region imaged in experiments where GCaMP was expressed using a pan-neuronal driver and CsChrimson was expressed with either the dPR1 or TN1A-2 driver. **f**, Pan-neuronal calcium imaging of the wing-related neuropils while activating dPR1. Frames were averaged across trials and flies (*n* = 6 trials for each irradiance; *n* = 7 flies), followed by further averaging across z-planes separately for dorsal and medial volumes of the VNC. The dashed circle in the image for the irradiance of 156 μW/mm^2^ from the dorsal volume highlights a region that showed strong activation during dPR1 but not TN1A-2 stimulation. **g**, Same as **f** but for TN1A-2 (*n* = 6 trials for each irradiance; *n* = 6 flies). **h**, dPR1 neurons in the dorsal and medial volumes visualized with the dPR1 split-Gal4 line. Similar expression patterns were observed in three flies for each genotype. **i**, Same as **h** but for TN1A-2 neurons visualized with the TN1A-2 split-Gal4 line.

To further examine the generality of nested activation during singing, we expressed jGCaMP7f with a pan-neuronal driver and performed volumetric calcium imaging in the wing-related neuropils (Fig. 4a)^40^, which are innervated by at least tens of cell types^11,14,20,22,23,34,27,28,29,41^, while inducing song with optogenetic stimulation of the song driver (Fig. 4b). Since two-photon microscopy cannot resolve individual neuronal processes within dense projections, the measured calcium signal in each voxel likely reflected the combined activity of multiple neurons. Optogenetic stimulation elicited an increase in calcium signals in a large proportion of the recorded volume (Fig. 4c and Supplementary Videos 3 and 4). A large fraction of the activated voxels showed comparable calcium signals during pulse and sine song (Fig. 4c,d,f and Extended Data Fig. 6a–d), suggesting that most of the song-related neurons are similarly active irrespective of song type. We also found that a subset of the activated voxels modulated calcium signals when the fly switched between pulse and sine song, with a majority of these modulated neurons preferring pulse song (Fig. 4c–g and Extended Data Fig. 6a–d). These observations suggest that the nested activity pattern observed in cell-type-specific recordings of dPR1 and TN1A-2 neurons is a general property of the song circuit during singing—one population of neurons is active both during pulse and sine song while another population is selectively active during pulse song.

The song driver might label neurons that are not related to singing, raising the possibility that optogenetic stimulation of these neurons contributed to voxel activation during both pulse and sine song. To test this possibility, we examined the patterns of pan-neuronal calcium signals induced by optogenetic stimulation of the dPR1 or TN1A-2 split-Gal4 drivers (Extended Data Fig. 6e), which cleanly label these cell types and induce pulse and sine song, respectively (Fig. 1b). Stimulation of either cell type led to widespread responses in largely overlapping volumes (Extended Data Fig. 6f,g), as expected from the neurons active during both pulse and sine song. Yet, the activation patterns induced by the stimulation of these cell types were not identical. For example, dPR1 but not TN1A-2 activation induced strong responses along the anterior–posterior axis around the midline in the wing tectulum (Extended Data Fig. 6f,g), in the same region where pulse-preferring voxels were observed during stimulation of the song driver (Fig. 4d and Extended Data Fig. 6a,b). These differential response patterns were not explained by calcium signals of the neurons experiencing optogenetic stimulation (that is, dPR1 and TN1A-2) because dPR1 and TN1A-2 neurons innervate highly overlapping regions (Extended Data Fig. 6h,i). These results support the idea that flexible song production involves two neural populations, one active during both pulse and sine song and the other active selectively during pulse song.

### Song-type selective VNC signals are produced by *fru* neurons

**Extended Data Fig. 7:**
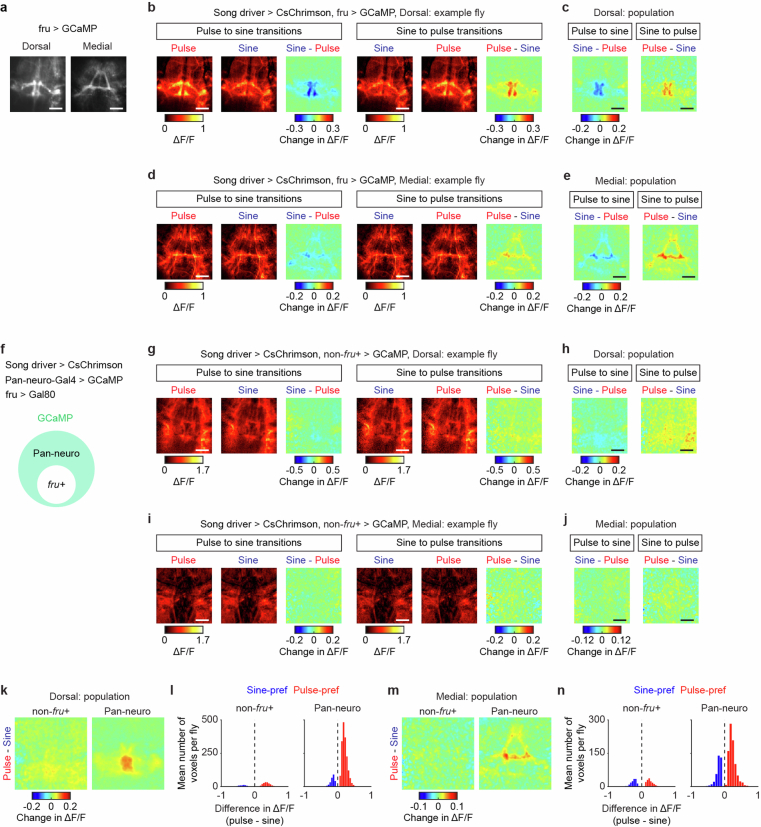
Song type selective signals in the circuit are carried by a genetically defined population of neurons. **a**, Example images of the calcium signals recoded from the dorsal (left) and medial (right) parts of the VNC. Images represent averages of frames across z-planes and time. Scale bars, 50 μm. **b**, Changes in calcium signals across song-type transitions for the example fly shown in Fig. 5b. Images were averaged across z-planes and time. Left, average frames during pulse to sine transitions (*n* = 137 transitions). Right, average frames during sine to pulse transitions (*n* = 65 transitions). Scale bars, 50 μm. **c**, Population averaged changes in ΔF/F during song-type transitions for the recordings from the dorsal part of the VNC for *fru*-expressing neurons (*n* = 6 flies). Scale bars, 50 μm. **d,e**, Same as **b,c** but for the recordings from the medial part. (**d**) n = 142 and 68 pulse-to-sine and sine-to-pulse transitions, respectively. (**e**) *n* = 5 flies. **f–n**, Calcium imaging of non-*fru*-expressing neurons. **f**, GCaMP was expressed using the pan-neuronal driver while its expression was suppressed in *fru*-expressing neurons. **g,h**, Same as **b,c** but for non-*fru*-expressing neurons. (**g**) *n* = 34 and 13 pulse-to-sine and sine-to-pulse transitions, respectively. (**h**) *n* = 5 flies. **i,j**, Same as **d,e** but for non-*fru*-expressing neurons. (**i**) *n* = 32 and 12 pulse-to-sine and sine-to-pulse transitions, respectively. (**j**) *n* = 3 flies. **k**, Population averaged ΔF/F for the difference between pulse and sine song around song-type transitions for the recordings from the dorsal part of the VNC for non-*fru*-expressing neurons (Left, *n* = 5 flies). The pan-neuronal imaging result described in Fig. 4d is also shown for comparison (Right). **l**, Histograms of the mean difference in ΔF/F between pulse and sine song during song-type transitions for voxels that changed ΔF/F depending on song-type (*P* < 0.001, two-sided permutation test). Left, non-*fru*-expressing neurons (*n* = in 5 flies). Right, the pan-neuronal imaging result shown in Fig. 4e. **m,n**, Same as **k,l** but for the recordings from the medial part (*n* = 3 flies).

During pan-neuronal imaging, the pulse-preferring voxels were localized to a region called the meso-thoracic triangle in the medial VNC^23^ and the medial and lateral parts of the dorsal VNC (Fig. 4d,f). We noticed that this distribution is similar to the innervation pattern of neurons expressing *fru* (refs. ^20,23^) (Extended Data Fig. 7a), a sex determination gene that regulates sexually dimorphic behaviors including courtship^42^. There are approximately 500 *fru*-expressing neurons in the male VNC, comprising at least 35 morphologically distinct cell types^20,23^. A subset of these cell types, including dPR1, are known to innervate a VNC region overlapping the pulse-preferring voxels^20,23^. Thus, these neurons might be the source of the pulse-selective signals observed in the pan-neuronal recording.

**Fig. 5:**
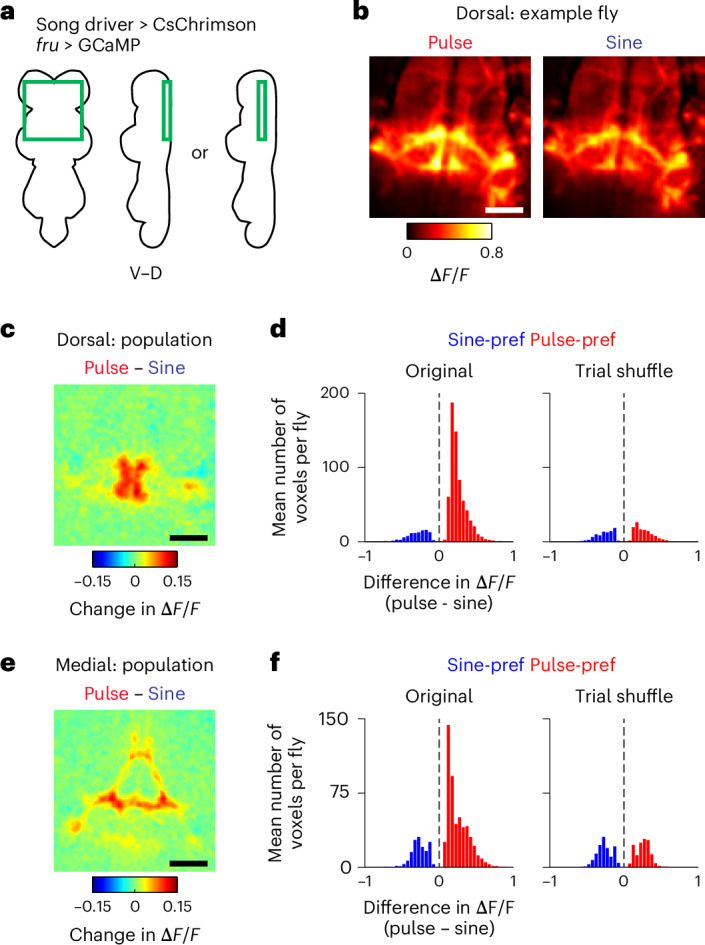
Song-type selective signals in the motor circuit are carried by a genetically defined population of neurons. **a**, Schematic of the imaged region. For each fly, images were obtained from either dorsal or medial regions of the VNC. CsChrimson and GCaMP were expressed using the song driver and *fru*-Gal4, respectively. **b**, The mean Δ*F*/*F* during pulse (left) and sine (middle) song around song-type transitions for the dorsal part of the VNC in an example fly (*n* = 202 transitions). Images were averaged across *z*-planes and time. Scale bar, 50 μm. **c**, Population-averaged Δ*F*/*F* for the difference between pulse and sine song around song-type transitions for the recordings from the dorsal part of the VNC (*n* = 6 flies). Scale bar, 50 μm. **d**, Histogram of the mean difference in Δ*F*/*F* between pulse and sine song during song-type transitions for the voxels that changed Δ*F*/*F* depending on song type (*P* < 0.001, two-sided permutation test). Data of the recordings from the dorsal part of the VNC were combined (*n* = 6 flies). A histogram expected by chance was obtained using a randomization method (‘Trial shuffle’; Methods). **e**,**f**, Same as **c** (**e**) and **d** (**f**) but for the recordings from the medial part of the VNC (*n* = 5 flies). See also Extended Data Fig. 7.

To test this possibility, we expressed jGCaMP7f in the *fru* neurons using the *fru*-Gal4 line^43^ and recorded calcium signals in the wing-related neuropils while inducing song by optogenetic activation of the song driver (Fig. 5a). We found that some voxels showed similar activation patterns during pulse and sine song (Fig. 5b,c,e and Extended Data Fig. 7b–e), suggesting that a population of *fru* neurons are similarly active irrespective of song type. We also found that some voxels modulated calcium signals depending on song type. Most of these voxels showed stronger signals during pulse than sine song (Fig. 5b–f and Extended Data Fig. 7b–e), indicating that some *fru* neurons are more active during pulse than sine song. The locations of these pulse-preferring voxels (Fig. 5c,e) matched those detected with pan-neuronal imaging (Fig. 4d,f). Thus, the activity of a population of *fru*-expressing neurons contributes to the pulse-selective signals observed during pan-neuronal imaging.

To examine the extent to which *fru*-expressing neurons account for the pulse-selective signals observed in pan-neuronal imaging, we took an intersectional genetic approach to express GCaMP in all non-*fru* neurons. We used a pan-neuronal driver to express Gal4, which drove the expression of jGCaMP7f, while suppressing jGCaMP7f expression in *fru* neurons with the Gal4 inhibitor, Gal80 (ref. ^23^) (Extended Data Fig. 7f). A large proportion of the imaged volume was similarly activated during pulse and sine song (Extended Data Fig. 7g–n), as in the pan-neuronal imaging (Fig. 4d–f and Extended Data Fig. 6a–d). However, we observed a profound reduction in the voxels selective for song type when jGCaMP7f expression was suppressed in *fru* neurons (Extended Data Fig. 7g–n). Notably, *fru* neurons comprise only a few percent of the neurons in the VNC^23,44^, indicating that these neurons have a disproportional impact on the pulse-selective signals. These results suggest that *fru*-expressing neurons play a key role in the production of pulse song.

### Singing involves nested activation of descending pathways

We next characterized descending signals provided from the brain to the VNC during song. Two types of descending neurons, pIP10 and pMP2, extensively innervate the wing-related neuropils^17,20,23,24^. Optogenetic activation of pIP10 induces both pulse and sine song^17,24^, whereas pMP2 activation induces pulse but not sine song^24^. These findings suggest that pIP10 and pMP2 play distinct roles in song production. However, it remains unclear whether and how these neurons are activated to pattern song sequences.

**Fig. 6:**
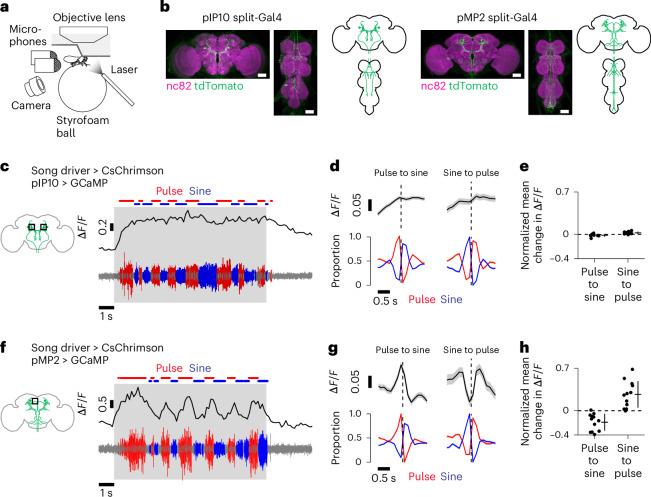
Calcium dynamics of parallel descending pathways during the singing of pulse and sine song. **a**, Schematic of calcium imaging in the brain during fly singing. **b**, Expression patterns of the pIP10 split-Gal4 (left) and the pMP2 split-Gal4 (right). The confocal stacks for the pIP10 split-Gal4 and pMP2 split-Gal4 were from ref. ^17^ and ref. ^24^, respectively. Similar expression patterns were observed in three flies for each genotype. Scale bars, 50 μm. **c**, Example Δ*F*/*F* trace of pIP10 neurons (top) together with the simultaneously recorded sound (bottom). Calcium signals reflect combined activity of the left and right pIP10 neurons. **d**, Time course of pIP10 Δ*F*/*F* (top) and song (bottom) around song-type transitions. Dashed vertical lines represent the timing of transitions. Data are from 9 flies and represented as mean ± s.e.m. across transitions for both Δ*F*/*F* and song (*n* = 2,827 and 1,145 events for pulse-to-sine and sine-to-pulse transitions, respectively). **e**, The mean change in Δ*F*/*F* after song-type transitions relative to Δ*F*/*F* before the transitions (see Methods for details) for pIP10. Each dot represents a neuron. Lines represent mean ± s.d. across neurons (*n* = 9 flies). *P* = 0.21 (pulse-to-sine transitions); *P* = 0.020 (sine-to-pulse transitions); two-sided one-sample *t*-test with Bonferroni correction. **f–h** Same as **c–e** but for pMP2. Calcium signals were recorded from neurites of individual pMP2 neurons. **g**, *n* = 1,487 (pulse-to-sine) and 524 (sine-to-pulse) transitions from 12 neurons in 7 flies. **h**, *n* = 12 neurons in 7 flies. *P* = 0.0012 (pulse-to-sine transitions); *P* = 0.0027 (sine-to-pulse transitions). See also Extended Data Fig. 8.

**Extended Data Fig. 8:**
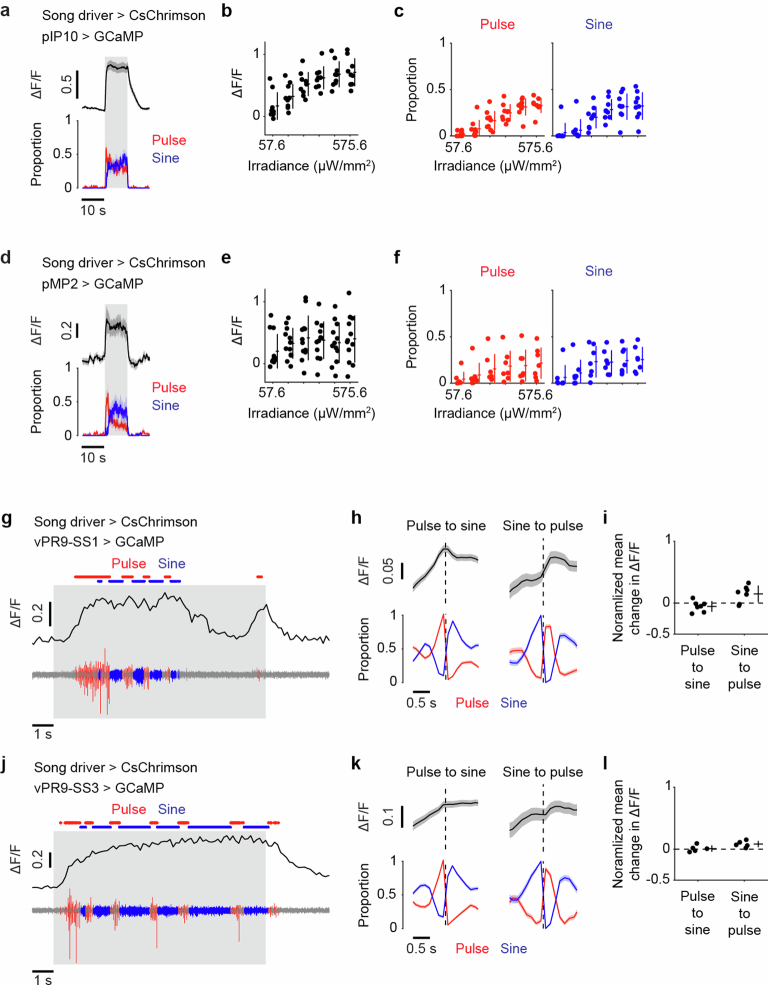
Calcium dynamics of parallel descending pathways during the singing of pulse and sine song. **a**, Time course of ΔF/F recorded from pIP10 (top) and the proportions of pulse and sine song (bottom) during laser stimulation at an irradiance of 575.6 μW/mm^2^. Data are represented as mean ± s.e.m. across neurons (*n* = 9 flies). **b**, The mean ΔF/F of pIP10 during the stimulation of the song driver. Each dot represents a fly from which the combined calcium signals of the left and right pIP10 neurons were recorded. Lines represent mean ± s.d. across flies (*n* = 9 flies). The X-axis is in a logarithmic scale. **c**, The mean proportions of pulse and sine song during the stimulation period in the recordings from pIP10. Each dot represents a fly. Lines represent mean ± s.d. across flies (*n* = 9 flies). **d–f**, Same as **a–c** but for pMP2 except that the activity of individual neurons was analyzed. (**d,e**) *n* = 12 neurons in 7 flies. (**f**) *n* = 7 flies. **g**, Example ΔF/F trace of vPR9 neurons labeled by the vPR9-SS1 line^24^ (top) together with the simultaneously recorded sound (bottom). Calcium signals were recorded from part of the neurite of vPR9 neurons. **h**, Time course of ΔF/F of the vPR9 neurons labeled by the vPR9-SS1 line (top) and song (bottom) around song-type transitions. Dashed vertical lines represent the timing of transitions. Data are from 7 flies and represented as mean ± s.e.m. across transitions for both ΔF/F and song (*n* = 384 and 134 events for pulse-to-sine and sine-to-pulse transitions, respectively). **i**, The mean change in ΔF/F after song-type transitions relative to ΔF/F before the transitions for the the vPR9 neurons labeled by the vPR9-SS1 line. Each dot represents a fly. Lines represent mean ± s.d. across flies (*n* = 7 flies). *P* = 0.32 (pulse-to-sine transitions); *P* = 0.053 (sine-to-pulse transitions); two-sided one-sample *t*-test with Bonferroni correction. **j–l**, Same as **g–i** but for vPR9 neurons labeled by the vPR9-SS3 line^24^. (**k**) Data are from 5 flies (*n* = 341 and 106 events for pulse-to-sine and sine-to-pulse transitions, respectively). (**l**) *n* = 5 flies. *P* = 1.00 (pulse-to-sine transitions); *P* = 0.046 (sine-to-pulse transitions).

To examine activity patterns of pIP10 and pMP2 during song, we expressed jGCaMP7f in one cell type at a time with split-Gal4 drivers^17,24^ and recorded calcium signals in the brain of the fly standing on a ball (Fig. 6a,b). Song was induced by optogenetic activation of the song driver. We found that pIP10 showed increased calcium signals during optogenetic activation as expected for a role in singing (Fig. 6c and Extended Data Fig. 8a–c). These neurons displayed similar levels of calcium signals during pulse and sine song, with a weak preference for pulse (Fig. 6c–e). These results suggest that pIP10 is active during both pulse and sine song.

Similar to pIP10, pMP2 showed increased calcium signals during optogenetic activation (Fig. 6f and Extended Data Fig. 8d–f). However, in contrast to pIP10, pMP2 signals were strongly selective for song type—calcium signals increased during pulse song and decreased during sine song (Fig. 6f–h). The pattern of calcium signal modulation was similar to the pattern observed for dPR1 (Fig. 1). These results suggest that pMP2 is active during pulse but not sine song. Taken together, parallel descending pathways exhibit nested activation patterns similar to those we observed for the VNC. One pathway, pIP10, is active irrespective of the song type the fly sings, whereas the other pathway, which includes pMP2, is active only during pulse song. These observations suggest that pIP10 neurons encode whether to sing or not, while the activity of pMP2 neurons contributes to specifying whether to sing pulse song.

A recent study suggested that vPR9 neurons, GABAergic neurons in the VNC, might play a role in switching between pulse and sine song because optogenetic activation of different vPR9 neurons preferentially suppresses pulse or sine song^24^. To test this hypothesis, we recorded calcium signals from pulse-suppressing and sine-suppressing vPR9 neurons (labeled by the vPR9-SS1 and vPR9-SS3 drivers, respectively^24^) while inducing song by optogenetic activation of the song driver. We found that both the pulse-suppressing and sine-suppressing populations were active during both pulse and sine song, with a weak preference for pulse (Extended Data Fig. 8g–l). The absence of a clear reciprocal pattern of activity in these two vPR9 cell types suggests that these neurons alone are unlikely to drive the switch between pulse and sine songs.

### Different cell types distinctly influence song patterning

**Fig. 7:**
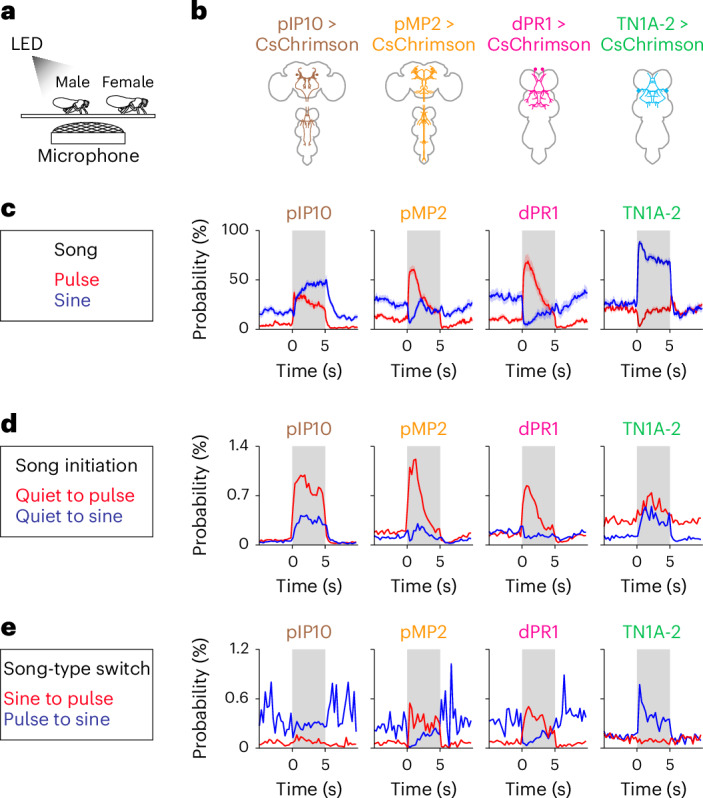
Optogenetic activation of different cell types distinctly influences the patterning of song sequences. **a**, Schematic of the paired fly optogenetic activation experiment. CsChrimson was expressed only in the male. **b**, Target cell types. **c**, Time course of the probabilities of pulse (red) and sine (blue) song induced by LED stimulation. The shaded area represents the period during which LED stimulation was applied. Data are represented as mean ± s.e.m. across flies (*n* = 12 flies for pIP10, pMP2 and dPR1; *n* = 11 flies for TN1A-2). **d**, Time course of the song initiation probabilities (red, quiet to pulse events; blue, quiet to sine events) during LED stimulation at the irradiance of 4.0 μW mm^−2^ for pIP10 and TN1A-2 and 11.9 μW mm^−2^ for pMP2 and dPR1. For each genotype, the probabilities were calculated by aggregating data from 12 flies (pIP10, pMP2 and dPR1) or 11 flies (TN1A-2). **e**, Same as **d** but for the song switch probabilities (red, sine to pulse events; blue, pulse to sine events). See also Extended Data Fig. 9.

**Extended Data Fig. 9:**
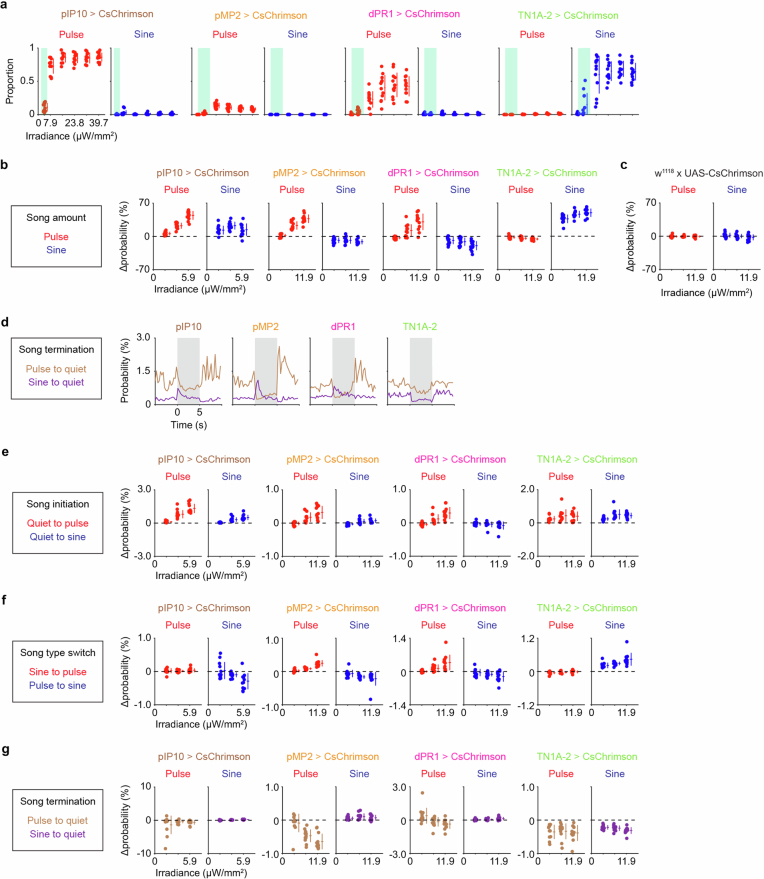
Optogenetic activation of different cell types distinctly influences the patterning of song sequences. **a**, The mean proportions of pulse (left) and sine (right) song during the stimulation period in the single fly optogenetic activation experiment. Each dot represents a fly. Lines represent mean ± s.d. across flies (*n* = 12 flies for each genotype). Shaded areas indicate the range of light intensities that were used in the paired fly optogenetic activation experiment. **b**, The mean difference in the proportions of pulse (left) and sine (right) song between the stimulation and 5-s pre-stimulation periods. Each dot represents a fly. Lines represent mean ± s.d. across flies (*n* = 12 flies). **c**, Same as **b** but for flies in a control genotype (*n* = 12 flies). **d**, Time course of the song termination probabilities (mustard, pulse to quiet events; purple, sine to quiet events) during LED stimulation at the irradiance of 4.0 μW/mm^2^ for pIP10 and TN1A-2 and 11.9 μW/mm^2^ for pMP2 and dPR1. For each genotype, the probabilities were calculated by aggregating data from 12 flies (pIP10, pMP2, and dPR1) or 11 flies (TN1A-2). **e–g**, The mean difference in the transition probabilities between the stimulation and 5-s pre-stimulation periods in the paired fly optogenetic activation experiment. Each dot represents a fly. Lines represent mean ± s.d. across flies (*n* = 11–12 flies). (**e**) The song initiation probabilities. (**f**) The song type switch probabilities. (**g**) The song termination probabilities.

Calcium imaging in singing flies revealed cell-type-specific activation patterns during pulse and sine song. To probe how the activity of each cell type contributes to patterning song sequences, we activated pIP10, pMP2, dPR1 and TN1A-2 neurons in the context of natural courtship. Male flies were paired with virgin females to induce song, while the activity of each cell type was boosted with optogenetics (Fig. 7a,b). To avoid excessive activations, which can be physiologically irrelevant, we used activation light intensities that induced song only moderately when applied in solitary flies (Extended Data Fig. 9a).

We found that activation of these cell types differentially affects the amount of pulse and sine song (Fig. 7c and Extended Data Fig. 9b,c), as shown previously^24^. pIP10 activation increased both pulse and sine song, consistent with the observation that these neurons are similarly active during both song types (Fig. 6). Activation of pMP2 and dPR1 neurons, the neurons active during pulse but not sine song (Figs. 1 and 6), increased pulse and decreased sine song. Activation of TN1A-2 neurons, which are active during both song types with a preference for sine (Fig. 1), increased sine song without changing the amount of pulse song. Thus, cell-type-specific activation effects on song amounts aligned with their calcium dynamics during song.

Changes in pulse and sine song amounts can arise from alterations in the probability of song initiation (quiet to pulse or sine song), song-type switch and/or song termination. We found that optogenetic activation influenced these probabilities in a cell-type-specific manner (Fig. 7d,e and Extended Data Fig. 9d–g). While activation of every cell type caused a change in the song initiation probability (Fig. 7d and Extended Data Fig. 9e), activation of pMP2, dPR1 and TN1A-2, but not pIP10, affected the song-type switch probability (Fig. 7e and Extended Data Fig. 9f). Notably, the changes in these probabilities were qualitatively different between the pulse-promoting neurons (pMP2 and dPR1) and the sine-promoting neurons (TN1A-2). For example, activation of pMP2 and dPR1 influenced both the sine-to-pulse and pulse-to-sine probabilities, whereas TN1A-2 activation specifically modified the pulse-to-sine probability (Fig. 7e and Extended Data Fig. 9f). These results suggest that the activity of the pulse-promoting and sine-promoting neurons makes distinct contributions to patterning song sequences with alternating pulse and sine songs.

### Connectome analysis reveals song circuit architecture

**Fig. 8:**
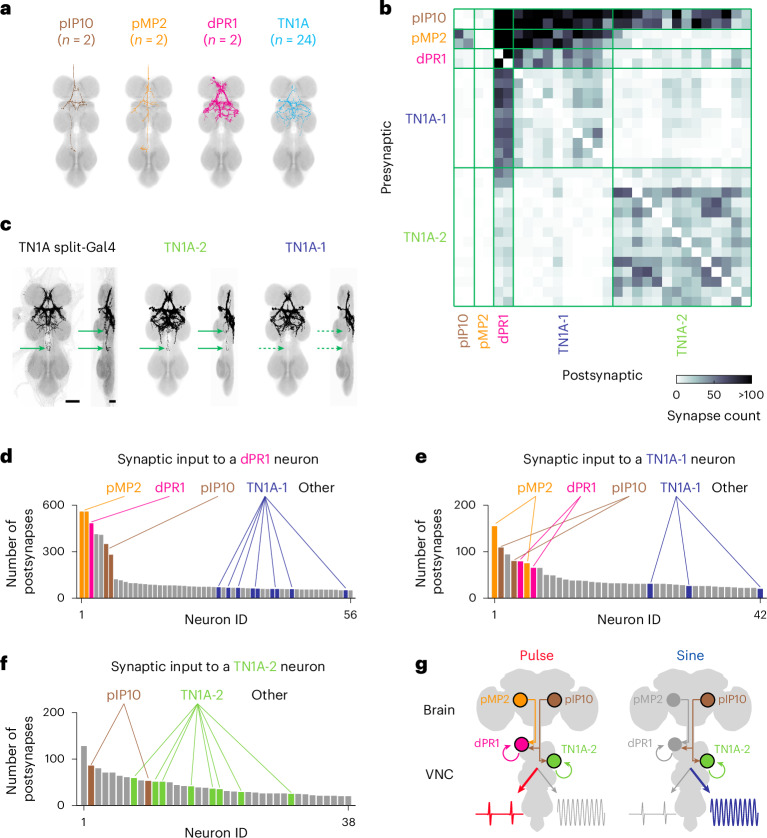
Neural circuit architecture for courtship song production. **a**, Electron microscopy (EM) reconstruction images of single pIP10, pMP2, dPR1 and TN1A neurons. **b**, Synaptic connectivity matrix among pIP10, pMP2, dPR1 and TN1A neurons. TN1A neurons can be separated into two populations (TN1A-1 and TN1A-2) based on connectivity. **c**, Expression pattern of the TN1A-2 split-Gal4 (left) and the overlaid EM reconstruction images of TN1A-2 (middle) and TN1A-1 (right) neurons. Arrows indicate the neurites that were present in the TN1A-2 split-Gal4 and TN1A-2 neuron images but not in TN1A-1 neuron images. Scale bars, 50 μm. **d**, Histogram of the number of synapses from upstream neurons onto a dPR1 neuron (body ID: 10300). Only neurons with 50 or more synapses onto dPR1 are shown. **e**,**f**, Same as **d** but for a TN1A-1 (body ID: 15521) (**e**) and a TN1A-2 (body ID: 13928) (**f**) neuron. **g**, Model of pulse and sine song production. See also Extended Data Fig. 10.

**Extended Data Fig. 10:**
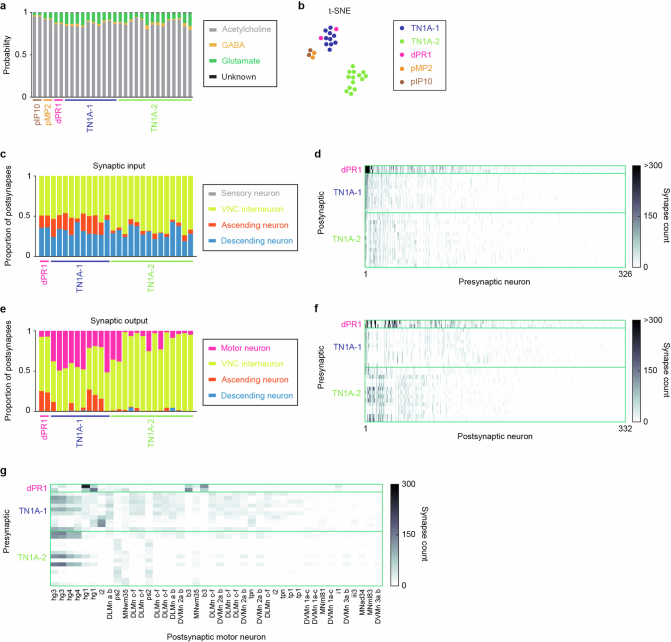
Neural circuit architecture for courtship song production. **a**, The probability of predicted neurotransmitter for each neuron of interest. Data are from the MANC connectome dataset^29^. **b**, A t-distributed stochastic neighbor embedding plot generated from the connectivity matrix in Fig. 8b. **c**, The proportion of the synaptic input from each category of presynaptic neurons. Data are plotted for individual dPR1, TN1A-1, and TN1A-2 neurons. Neuron category data are from the MANC connectome dataset^29^. **d**, The heatmap showing the presynaptic input to individual dPR1, TN1A-1, and TN1A-2 neurons. **e,f**, Same as **c,d** but for synaptic output from dPR1, TN1A-1, and TN1A-2 neurons. **g**, The heatmap showing the synaptic output to motor neurons from dPR1, TN1A-1, and TN1A-2 neurons.

To examine how nested activity patterns in the VNC are driven by descending input, we analyzed the synaptic connectivity among pIP10, pMP2, dPR1 and TN1A neurons using the connectome of the male VNC^27,28,29^. All of these neurons are cholinergic^24^ and are likely to be excitatory. We identified these neurons in the connectome dataset based on morphological characteristics. We found only two neurons, one per hemisphere, for pIP10, pMP2 and dPR1, as reported previously^17,20,23,24^ (Fig. 8a). There were 24 neurons whose morphology matched TN1A (ref. ^22^) (Fig. 8a). As expected, a neurotransmitter classification algorithm for electron microscopy images predicted that all of these neurons in the electron microscopy dataset are cholinergic^29,45^ (Extended Data Fig. 10a).

We first mapped the connectivity among these neurons and found that TN1A neurons can be separated into two groups, referred to as TN1A-1 and TN1A-2 (Fig. 8b and Extended Data Fig. 10b). As mentioned earlier, the TN1A split-Gal4 line we used in previous experiments is likely to target TN1A-2 neurons because the driver line labels neurites that were present only in the TN1A-2 population (Fig. 8c). These two types of TN1A neurons, as well as dPR1 neurons, are connected to hundreds of neurons (Extended Data Fig. 10c–g). Below, we focus on whether and how the connectivity among the cell types we studied is related to their dynamics during song.

Both of the descending neurons, pMP2 and pIP10, provide many synaptic inputs to dPR1 (Fig. 8b,d). dPR1 and pMP2 neurons are active selectively during pulse song (Figs. 1 and 6), suggesting that the descending neuron pMP2 carries pulse-selective signals to dPR1. In contrast, the descending neuron pIP10 was active during both pulse and sine song (Fig. 6). These observations of neuron activity and connectivity are consistent with a model in which concurrent input from pIP10 and pMP2 contributes to activation of dPR1 neurons. Since optogenetic activation of pIP10 alone can drive pulse song, we infer that artificially high levels of pIP10 activity can drive dPR1 activity without pMP2 input.

Similar to dPR1, TN1A-1 neurons receive many inputs from pIP10 and pMP2 (Fig. 8b,e). Furthermore, dPR1 and TN1A-1 neurons are interconnected with each other (Fig. 8b,e). These results suggest that TN1A-1 and dPR1 neurons would exhibit similar activity patterns. While specific driver lines for TN1A-1 neurons are not available yet, the pulse-preferring TN1 neurons we studied with *dsx*-Gal4 might include TN1A-1 neurons because their activity patterns resembled those of dPR1 (Fig. 3).

TN1A-2 neurons receive substantial input from pIP10 but not from pMP2 or dPR1 (Fig. 8b,f). The connections within the TN1A-2 population are generally stronger than those between TN1A-1 and dPR1 neurons (Fig. 8b,f). Thus, activity patterns of TN1A-2 neurons may differ from those of TN1A-1 and dPR1 neurons. Indeed, the TN1A neurons labeled by the driver line, which are likely TN1A-2 neurons, exhibit activity patterns more similar to those of pIP10 than to dPR1 or pMP2 (Figs. 1 and 6). Together, these results suggest that the nested activity patterns in the VNC during pulse and sine song are largely inherited from parallel descending pathways (Fig. 8g).

Further connectome analysis revealed that dPR1 and TN1A neurons are embedded in networks of other interneurons in the VNC. The majority of inputs to dPR1 and TN1A neurons are from VNC interneurons (Extended Data Fig. 10c,d), and, in turn, dPR1 and TN1A neurons send output primarily to VNC interneurons (Extended Data Fig. 10e,f). Both cell types also provide output to wing motor neurons in a cell-type-specific manner (Extended Data Fig. 10g). The connectivity to motor neurons varies even between members of the same cell type (Extended Data Fig. 10g), suggesting that there is heterogeneity of motor functions among neurons of the same cell type.

## Discussion

Studies of motor circuits during naturalistic behavior, in both invertebrates and vertebrates, have often been hampered by the inability to monitor the relevant circuits in behaving animals. To overcome this limitation, we developed a two-photon calcium imaging assay for recording neural activity from the VNC of *Drosophila* males while they performed moment-to-moment switching between two types of courtship song, pulse and sine. We found that sine song involves activation of a population of VNC neurons that are also active during pulse song, whereas pulse song involves activation of additional VNC neurons. A population of song-related descending neurons also exhibit the nested activation patterns associated with song types. Connectomic analysis revealed strong synaptic connections between descending and VNC neurons that showed similar activation patterns, consistent with a model in which parallel descending pathways drive overlapping yet distinct motor modules. These findings suggest that activation of nested populations of neurons, spanning from the brain to the VNC, orchestrates distinct acoustic communication signals during courtship.

Previous studies showed that manipulation of specific neurons in the VNC often causes changes in the amount of either pulse or sine song^22,24^, leading to the proposal that different types of song are produced by selectively activating independent neural populations^19^. In line with this model, dPR1 neurons, which induce pulse song upon artificial activation, showed increased activity during pulse but not sine song (Fig. 1). However, contrary to the model, TN1A-2 neurons are active during both pulse and sine song although these neurons induce only sine song when artificially activated (Fig. 1). Consistent with their activation during pulse song, we recently found that silencing the activity of TN1A-2 neurons influences the amplitude of pulse song as well as the amount of sine song^24^. Recordings from broader populations of VNC neurons further demonstrated that this nested activation pattern is a general feature of the song motor system—some neurons are active during both types of song and most of the song-type selective neurons prefer pulse song. This is in stark contrast with a recently proposed model where pulse and sine songs result from the selective activation of pulse-driving and sine-driving neurons, whose selectivity is realized through mutual inhibition and rebound excitation^19^. This model was developed partially based on the observation that optogenetic activation of pIP10 neurons leads to activation of certain TN1 neurons and inhibition of others^19^. However, it is unclear whether these activities are related to singing. We found that virtually all TN1 neurons showed excitatory responses to pIP10 stimulation during the singing of pulse song (Extended Data Fig. 5). Sensory feedback from the wings is not likely to account for the absence of inhibition in sine-driving neurons (Extended Data Fig. 4). Although the factors responsible for the varied TN1 activity patterns in different experimental conditions remain unidentified, our direct recording of neural activity during singing demonstrates that the singing of pulse and sine songs involves activation of overlapping populations of VNC neurons.

It was shown previously that a population of wing control muscles are active during both pulse and sine song, as we observe for TN1A-2 neurons^10^, suggesting that TN1A-2 neurons shape the pattern of activity in these muscles. These muscles have been proposed to control features of wing movement relevant for both pulse and sine song, such as the angle of attack^10,11^. Indeed, silencing wing motor neurons for some of these muscles alters properties of both types of song^11^. Thus, pulse song and sine song involve activation of a shared population of VNC neurons and wing muscles that may shape wing oscillations common to both song types.

In contrast to TN1A-2, dPR1 neurons are active during pulse but not sine song. Similarly, a group of wing control muscles are active during pulse but not sine song^10^, suggesting that these muscles are controlled by dPR1 neurons. These muscles are thought to contribute to the damping of wing oscillations during inter-pulse intervals as well as the initiation of wing movement at the end of each inter-pulse interval^10,11^. While some wing muscles are active during both song types and some are selective for pulse song, no wing muscle is specifically active during sine song^10,11,31^. This suggests that a group of muscles generate wing oscillations during both pulse and sine song while additional muscles are recruited during pulse song to shape inter-pulse intervals. We propose that TN1A-2 and dPR1 neurons play key roles in these two motor functions.

Connectome analysis revealed that dPR1 and TN1A-2 neurons are members of larger networks of VNC interneurons. The major output targets of both cell types are VNC interneurons (Extended Data Fig. 10), suggesting that dPR1 and TN1A-2 neurons generate patterned motor neuron activation through other interneurons. Indeed, our recent work showed that there are at least four other VNC cell types that play key roles in singing^24^. Notably, activity manipulation of these cell types also suggested that the network for sine song is contained within the network for pulse song^24^, providing additional support for a nested architecture of neural circuits for pulse and sine song. Because the VNC song circuit is capable of modulating specific song parameters, such as the amplitude of pulse song depending on the distance to females^16^, it is likely that the pulse and sine circuits consist of further functional modules. Indeed, manipulating the activity of different VNC cell types alters different pulse song parameters^20,24^. Similarly, silencing different wing motor neurons causes changes in distinct aspects of pulse song^11,14^. Thus, the song circuit may be composed of subnetworks that control specific song features through activation of distinct muscles.

Courting males modulate the proportions of pulse and sine songs based on the behavioral context defined by the distance to females and/or the males’ own locomotion^7,19^. We found that the descending neuron pMP2 is selectively active during pulse song and provides primary input to the pulse-promoting VNC neurons dPR1, suggesting that pMP2 plays a key role in context-dependent song selection. This proposal contrasts with a recent model by Roemschied et al.^19^. In their model, the activity of descending neurons is modulated depending on the context but not the song type being sung, and constant input from the descending neurons enables the pulse-driving and sine-driving VNC neurons to exhibit song-type selective activities. Our finding indicates that song-type selection, at least in part, is shaped by descending input from the brain.

In contrast to pMP2, the descending neuron pIP10 was active during both pulse and sine song and provides input to both dPR1 and TN1A-2. Consistent with their activity pattern, pIP10 neurons are causally involved in production of both pulse and sine song^17,24^. Artificial activation of pIP10 in solitary males induces primarily pulse song^17,24^, and strong artificial activation in the presence of females specifically increases pulse song^24^. These findings suggest that while pIP10 neurons can specifically drive pulse song in certain experimental conditions, their activity similarly promotes pulse and sine songs during natural courtship. This in turn suggests that pIP10 activity during natural courtship is not enough to induce activation of dPR1 and thus pulse song. Strong dPR1 activation, and thus pulse song, might require additional input from pMP2. pIP10 input may set the membrane potential of dPR1 close to the spiking threshold, thereby enabling dPR1 to rapidly respond to fluctuating pMP2 input for flexible song-type switching. Thus, descending neurons innervating the song motor system may form parallel pathways that independently specify when to sing a song, through pIP10 activity, and what song to sing, through differential activation of pMP2.

Production of diverse movements by nested activity patterns is not unique to the *Drosophila* song system. For example, in the mammalian respiratory system, a group of neurons are active during fictive normal breathing, whereas a subset of these active neurons become inactive during fictive gasping^46^. Notably, normal breathing and gasping are likely to involve activation of overlapping sets of muscles^3^, similar to the production of pulse and sine songs^10,31^. In contrast, movements driven by independent sets of muscles, as in the case of alternating movements of left and right limbs during locomotion^2^, may be controlled through separate neural populations. Thus, the pattern of muscle recruitment required for distinct movements may bias evolution toward either combinatorial or independent neural control of muscles.

In summary, we uncovered the functional circuit architecture for producing distinct courtship songs in *D. melanogaster*. Our calcium imaging assay in courting flies complements existing strengths of this system such as cell-type-specific drivers, activity manipulation^11,17,20,22,24^ and comprehensive circuit analysis with the connectome^27,28,29^. Together, the *Drosophila* song system will provide a unique opportunity to investigate the circuit mechanisms of neural computations underlying action selection and motor pattern generation.

## Methods

### Fly husbandry

Flies (*D. melanogaster*) were raised on cornmeal–molasses food at 23 °C under a 12-h/12-h light–dark cycle until eclosion. In all experiments, we used 5–7-d-old adult male flies collected within 6 h after eclosion and maintained in isolation in the dark. For the adult male flies used in experiments involving optogenetic stimulation, the food was supplemented with 0.4 mM all-*trans*-retinal (Toronto Research Chemicals). Virgin female flies used in behavioral experiments were housed in groups of approximately 20.

### Fly stocks and genotypes

Fly stocks used in this study are as follows: w^1118^ (Stern Laboratory); UAS-CsChrimson-tdTomato(VK00005) (ref. ^30^) (a gift from Gerald Rubin); VT037566-p65ADZp(attP40) (refs. ^47,48^) (Dickson Laboratory); VT041688-ZpGDBD(attP2) (ref. ^48^) (Bloomington Drosophila Stock Center (BDSC) cat. no. 72408); VT042732-p65ADZp(attP40) (ref. ^48^) (BDSC cat. no. 71534); dsx-ZpGDBD (ref. ^22^); UAS-CsChrimson-mVenus(attP18) (ref. ^30^) (BDSC cat. no. 55134); R22D03-LexA(attP2) (ref. ^26^) (a gift from Gerald Rubin); LexAop2-CsChrimson-tdTomato(VK00005) (ref. ^30^) (a gift from Vivek Jayaraman); UAS-IVS-jGCaMP7f(su(Hw)attP5) (ref. ^37^) (BDSC cat. no. 80906); UAS-IVS-myr::tdTomato(attP40) (ref. ^26^) (BDSC cat. no. 32222); w^1118^,hsFLPG5.PEST(attP3);;UAS-FRT.stop-myr::smGdP-HA(VK00005),UAS-FRT.stop-myr::smGdP-V5-THS-UAS-FRT.stop-myr::smGdP-FLAG(su(Hw)attP1) (ref. ^49^) (BDSC cat. no. 64085); VT040556-p65ADZp(attP40) (refs. ^17,48^) (BDSC cat. no. 72060); VT040347-ZpGDBD(attP2) (refs. ^17,48^) (BDSC cat. no. 75302); VT040347-ZpLexADBD(attP2) (ref. ^48^) (Dickson Laboratory); UAS-IVS-jGCaMP7s(su(Hw)attP5) (ref. ^37^) (BDSC cat. no. 80905); dsx-Gal4 (ref. ^39^) (a gift from Bruce Baker); R57C10-LexA(attP40) (ref. ^26^) (BDSC cat. no. 52817); UAS-CsChrimson-tdTomato(su(Hw)attP5) (ref. ^30^) (a gift from Vivek Jayaraman); LexAop2-IVS-jGCaMP7s(VK00005) (ref. ^37^) (BDSC cat. no. 80913); R57C10-Gal4(attP2) (ref. ^50^) (BDSC cat. no. 39171); fru-Gal4 (ref. ^43^)(BDSC cat. no. 66696); fru-Flp (ref. ^23^); tubP-FRT.stop-Gal80;MKRS/TM6B (ref. ^51^) (BDSC cat. no. 38878); R57C10-Gal4(attP40) (ref. ^50^) (a gift from Gerald Rubin); VT026873-p65ADZp(attP40) (ref. ^48^) (BDSC cat. no. 86831); VT028160-ZpGdbd(attP2) (ref. ^48^) (BDSC cat. no. 73842); VT019728-p65ADZp(attP40) (ref. ^48^) (Dickson Laboratory); VT025981-ZpGDBD(attP2) (ref. ^48^) (Dickson Laboratory); VT055068-p65ADZp(attP40) (ref. ^48^) (Dickson Laboratory); VT019739-ZpGDBD(attP2) (ref. ^48^) (Dickson Laboratory).

The dPR1, TN1A-2, pMP2, vPR9-SS1 and vPR9-SS3 split-Gal4 drivers were generated from the following split lines^24^: dPR1 from SS65789 (VT037566-p65ADZp(attP40);VT041688-ZpGDBD(attP2)); TN1A-2 from SS59832 (VT042732-p65ADZp(attP40);dsx-ZpGDBD/TM6b); pMP2 from SS43275 (VT026873-p65ADZp(attP40);VT028160-ZpGDBD(attP2)); vPR9-SS1 from SS61083 (VT019728-p65ADZp(attP40);VT025981-ZpGDBD(attP2)); vPR9-SS3 from SS58626 (VT055068-p65ADZp(attP40);VT019739-ZpGDBD(attP2)). These lines were identified by screening split lines identified by color depth maximum intensity projection search^52^ of candidate *fru*-expressing neurons and neurons identified with *trans*-Tango^53^ applied to a pIP10 split-Gal4 line^17,24^.

The genotypes of the flies used in each figure are as follows. Figure 1a and Extended Data Fig. 1j,o, w^1118^/Y;;UAS-CsChrimson-tdTomato(VK00005)/+; Figs. 1b and 8c and Extended Data Figs. 1a,c,d and 6h,i, w^1118^,UAS-CsChrimson-mVenus(attP18)/Y;VT037566-p65ADZp(attP40)/+;VT041688-ZpGDBD(attP2)/+, w;VT042732-p65ADZp(attP40)/+;dsx-ZpGDBD/UAS-CsChrimson-tdTomato(VK00005); Figs. 1c,d and 7 and Extended Data Figs. 1f–i and 9, VT037566-p65ADZp(attP40)/+;VT041688-ZpGDBD(attP2)/UAS-CsChrimson-tdTomato(VK00005), VT042732-p65ADZp(attP40)/+;dsx-ZpGDBD/UAS-CsChrimson-tdTomato(VK00005); Fig. 1f and Extended Data Fig. 1m–p, UAS-IVS-jGCaMP7f(su(Hw)attP5),UAS-IVS-myr::tdTomato(attP40)/+;R22D03-LexA(attP2),LexAop2-CsChrimson-tdTomato(VK00005)/+; Fig. 1g–j and Extended Data Figs. 3a–g,p and 5a,b, UAS-IVS-jGCaMP7f(su(Hw)attP5),UAS-IVS-myr::tdTomato(attP40)/VT037566-p65ADZp(attP40);R22D03-LexA(attP2),LexAop2-CsChrimson-tdTomato(VK00005)/VT041688-ZpGDBD(attP2); Fig. 1k–m and Extended Data Figs. 3h–m,q and 5b, UAS-IVS-jGCaMP7f(su(Hw)attP5),UAS-IVS-myr::tdTomato(attP40)/VT042732-p65ADZp(attP40);R22D03-LexA(attP2),LexAop2-CsChrimson-tdTomato(VK00005)/dsx-ZpGDBD; Extended Data Fig. 1b, w^1118^,hsFLPG5.PEST(attP3)/Y;VT037566-p65ADZp(attP40)/+;UAS-FRT.stop-myr::smGdP-HA(VK00005),UAS-FRT.stop-myr::smGdP-V5-THS-UAS-FRT.stop-myr::smGdP-FLAG(su(Hw)attP1)/VT041688-ZpGDBD(attP2); Extended Data Fig. 1e, w^1118^,hsFLPG5.PEST(attP3)/Y; VT042732-p65ADZp(attP40)/+;UAS-FRT.stop-myr::smGdP-HA(VK00005),UAS-FRT.stop-myr::smGdP-V5-THS-UAS-FRT.stop-myr::smGdP-FLAG(su(Hw)attP1)/dsx-ZpGDBD; Extended Data Fig. 1j, w^1118^/Y;VT037566-p65ADZp(attP40)/+;VT041688-ZpGDBD(attP2)/+, w^1118^/Y;VT042732-p65ADZp(attP40)/+;dsx-ZpGDBD/+; Extended Data Fig. 1k,l, R22D03-LexA(attP2)/LexAop2-CsChrimson-tdTomato(VK00005); Extended Data Fig. 3n, VT037566-p65ADZp(attP40),UAS-CsChrimson-tdTomato(su(Hw)attP5)/UAS-jGCaMP7f(su(Hw)attP5);VT041688-ZpGDBD(attP2),LexAop2-IVS-jGCaMP7s(VK00005)/+; Extended Data Fig. 3o, VT042732-p65ADZp(attP40),UAS-CsChrimson-tdTomato(su(Hw)attP5)/UAS-jGCaMP7f(su(Hw)attP5);dsx-ZpGDBD/+; Fig. 2a and Extended Data Fig. 4a, VT040556-p65ADZp(attP40),UAS-IVS-jGCaMP7s(su(Hw)attP5)/VT040556-p65ADZp(attP40);VT040347-ZpLexADBD(attP2),LexAop2-CsChrimson-tdTomato(VK00005)/VT040347-ZpGDBD(attP2); Fig. 2b and Extended Data Fig. 4b, VT040556-p65ADZp(attP40)/+;VT040347-ZpLexADBD(attP2)/LexAop2-CsChrimson-tdTomato(VK00005); Fig. 2c–e and Extended Data Figs. 4e–g,l and 5f–k, VT040556-p65ADZp(attP40),UAS-IVS-jGCaMP7s(su(Hw)attP5)/+;VT040347-ZpLexADBD(attP2),LexAop2-CsChrimson-tdTomato(VK00005)/dsx-Gal4; Fig. 2f–h and Extended Data Fig. 4h–j,m, VT040556-p65ADZp(attP40),UAS-IVS-jGCaMP7s(su(Hw)attP5)/VT042732-p65ADZp(attP40);VT040347-ZpLexADBD(attP2),LexAop2-CsChrimson-tdTomato(VK00005)/dsx-ZpGDBD; Fig. 7 and Extended Data Figs. 4c and 9, VT040556-p65ADZp(attP40)/+;VT040347-ZpGDBD(attP2)/UAS-CsChrimson-tdTomato(VK00005); Extended Data Fig. 4d, w^1118^/Y;;LexAop2-CsChrimson-tdTomato(VK00005)/+, w^1118^/Y;VT040556-p65ADZp(attP40)/+;VT040347-ZpLexADBD(attP2)/+; Fig. 3 and Extended Data Fig. 5c–e, UAS-IVS-jGCaMP7f(su(Hw)attP5),UAS-IVS-myr::tdTomato(attP40)/+;R22D03-LexA(attP2),LexAop2-CsChrimson-tdTomato(VK00005)/dsx-Gal4; Fig. 4c–g and Extended Data Figs. 6a–d and 7k–n, UAS-IVS-jGCaMP7f(su(Hw)attP5),UAS-IVS-myr::tdTomato(attP40)/+;R22D03-LexA(attP2),LexAop2-CsChrimson-tdTomato(VK00005)/R57C10-Gal4(attP2); Extended Data Fig. 6f,g, VT037566-p65ADZp(attP40),UAS-CsChrimson-tdTomato(su(Hw)attP5)/R57C10-LexA(attP40);VT041688-ZpGDBD(attP2),LexAop2-IVS-jGCaMP7s(VK00005)/+, VT042732-p65ADZp(attP40),UAS-CsChrimson-tdTomato(su(Hw)attP5)/R57C10-LexA(attP40);dsx-ZpGDBD/LexAop2-IVS-jGCaMP7s(VK00005); Fig. 5b–f and Extended Data Fig. 7a–e, UAS-IVS-jGCaMP7f(su(Hw)attP5),UAS-IVS-myr::tdTomato(attP40)/+;R22D03-LexA(attP2),LexAop2-CsChrimson-tdTomato(VK00005)/fru-Gal4; Extended Data Fig. 7g–n, UAS-IVS-jGCaMP7f(su(Hw)attP5)/R57C10-Gal4(attP40),tubP-FRT.stop-Gal80;R22D03-LexA(attP2),LexAop2-CsChrimson-tdTomato(VK00005)/fru-Flp; Fig. 6c–e and Extended Data Fig. 8a–c, UAS-IVS-jGCaMP7f(su(Hw)attP5),UAS-IVS-myr::tdTomato(attP40)/VT040556-p65ADZp(attP40);R22D03-LexA(attP2),LexAop2-CsChrimson-tdTomato(VK00005)/VT040347-ZpGDBD(attP2); Fig. 6f–h and Extended Data Fig. 8d–f, UAS-IVS-jGCaMP7f(su(Hw)attP5),UAS-IVS-myr::tdTomato(attP40)/VT026873-p65ADZp(attP40);R22D03-LexA(attP2),LexAop2-CsChrimson-tdTomato(VK00005)/VT028160-ZpGDBD(attP2); Extended Data Fig. 8g–i, UAS-IVS-jGCaMP7f(su(Hw)attP5)/VT019728-p65ADZp(attP40);R22D03-LexA(attP2),LexAop2-CsChrimson-tdTomato(VK00005)/VT025981-ZpGDBD(attP2); Extended Data Fig. 8j–l, UAS-IVS-jGCaMP7f(su(Hw)attP5)/VT055068-p65ADZp(attP40);R22D03-LexA(attP2),LexAop2-CsChrimson-tdTomato(VK00005)/VT019739-ZpGDBD(attP2); Fig. 7 and Extended Data Fig. 9, VT026873-p65ADZp(attP40)/+;VT028160-ZpGDBD(attP2)/UAS-CsChrimson-tdTomato(VK00005).

### Behavioral experiments

Song recordings in freely moving flies were conducted using the multi-channel song recording system described previously^54^. Briefly, the system is composed of 5-mm behavioral chambers, each equipped with a microphone (cat. no. CMP5247TF-K, CUI Devices). The output of the microphones was recorded at 5 kHz. Temperature and relative humidity were maintained at 23 °C and 50%, respectively. For optogenetic stimulation, an array of red light-emitting diodes (630 nm) (cat. no. NFLS-R300X3-WHT-LC2, Super Bright LEDs) was placed approximately 15 mm from the top of the chambers. During the recordings, the chambers were constantly illuminated with an ambient blue light (1.4 μW mm^−2^) (cat. no. STN-BBLU-A3A-10B5M-12V, Super Bright LEDs) to enable flies to perform visually guided behaviors such as orientation toward females. Data collection was conducted with MATLAB (2016b).

Flies were transferred from culture vials to the chambers with an aspirator without anesthesia. For the single fly optogenetic activation experiment, one male fly was placed in each chamber. For each trial, continuous light stimulation at a fixed power was applied for 10 s with an inter-trial interval of 20 s. Light power varied from trial to trial (6 levels between 4.0 and 39.7 μW mm^−2^) in a randomized order. Stimulation of each light power was repeated in six trials. For the paired fly optogenetic activation experiment, one male and one virgin female fly were placed in each chamber. For each trial, constant light stimulation, whose power varied from trial to trial (2.0, 4.0 or 5.9 μW mm^−2^ for pIP10 neurons and 4.0, 7.9 or 11.9 μW mm^−2^ for pMP2, dPR1 and TN1A-2 neurons), was applied for 5 s with an inter-trial interval of 10 s. Stimulation of the same light power was applied in 18–30 trials.

### Calcium imaging

Flies were prepared for calcium imaging in the VNC using the protocol described previously^55^ with modifications (Extended Data Fig. 2). Briefly, flies were placed on a Peltier plate that maintained temperature at 4 °C. The proboscis was fixed to the head capsule with ultraviolet glue, and the middle and hind legs were cut around the femur–trochanter junctions. The flies were then attached to a custom holding plate upside down (Fig. 1e) by applying the glue to the coxa and trochanter of the middle legs and femur of the forelegs. The thorax was largely free from the glue so as not to disturb thorax oscillations and thus wing movement. The cuticles and apodemes covering the pro- and meso-thoracic ganglia were removed with fine forceps. The dissection was performed in saline (103 mM NaCl, 3 mM KCl, 5 mM N-tris(hydroxymethyl) methyl-2aminoethane-sulfonic acid, 8 mM trehalose, 10 mM glucose, 26 mM NaHCO_3_, 1 mM NaH_2_PO_4_, 1.5 mM CaCl_2_ and 4 mM MgCl_2_; pH, 7.1–7.3; osmolarity, 270–275 mOsm) bubbled with carbogen (95% O_2_ and 5% CO_2_). Calcium imaging in the brain was performed using the same protocol as described before^55^ except that legs were not removed.

Calcium imaging was conducted using a two-photon microscope (Bergamo II, Thorlabs) with a pulsed laser tuned to 940 nm (InSight X3, Spectra-Physics). Data collection was performed with MATLAB (2019a) and ScanImage (2019a). The laser power measured under the objective lens was kept below 25 mW. A piezo actuator (cat. no. PFM450E, Thorlabs) was used to move an objective lens (cat. no. N16XLWD-PF, Nikon) for data acquisition from multiple *z*-planes. For experiments with optogenetic activation of pIP10, dPR1 or TN1A-2, two-dimensional images (512 × 512 pixels, pixel size 0.49 μm) were taken from 30 *z*-planes (4-μm step) at 1.4 volumes per s. For experiments with optogenetic activation of the song driver, images (256 × 256 pixels, pixel size 0.25–0.99 μm) were taken from 10 *z*-planes (4-μm step) at 7.1 volumes per s. In the recording from single dPR1 neurons, imaged volumes contained cell bodies of these neurons in both hemispheres in most of the experiments. In the recording from single TN1A-2 and TN1 neurons, imaged volumes contained cell bodies of most of the target neurons in one hemisphere. In the recording from pIP10 and pMP2 neurons, imaged volumes contained the neurites of these neurons in a dorsal part of the brain. In a subset of recordings where neurons expressed tdTomato in addition to GCaMP, signals of both fluorescent proteins were recorded simultaneously. Carbogenated saline was perfused throughout the recording.

During calcium imaging in the VNC, optogenetic activation light (660 nm) (cat. no. S1FC660, Thorlabs) passed through a long-pass filter (cat. no. FEL0650, Thorlabs) and was delivered with a patch cable (cat. no. M125L01, Thorlabs) placed at 6 mm away from the fly. The long-pass filter was not used in other calcium imaging experiments. For each fly, a continuous light stimulation at a fixed power was applied for 10 s with an inter-trial interval of 20 s. The light power varied from trial to trial (6 levels, 4.9–156.2 μW mm^−2^ for pIP10, dPR1 and TN1A-2 stimulations and recording in the brain, 4.9–17.1 μW mm^−2^ for song driver stimulation during recording in the VNC) in a randomized order. Stimulation of the same power was repeated in six trials for song driver stimulation, three trials for pIP10 stimulation in the analysis of neural activity during pulse song (Fig. 2 and Extended Data Fig. 4e–j), two trials both before and after cutting the wings in the wing cut experiment (Extended Data Fig. 4k–m), two trials in the experiment where both CsChrimson and jGCaMP7f were expressed in dPR1 or TN1A-2 neurons (Extended Data Fig. 3n,o) and six trials for dPR1 and TN1A-2 stimulations with pan-neuronal imaging (Extended Data Fig. 6e–g). For the pIP10 and song driver simulations in tethered flies without calcium imaging, the light power ranged from 2.4 to 14.6 μW mm^−2^. The same stimulus was presented in six trials.

The sound of vibrating wings was recorded with a pair of microphones (cat. no. NR-23158, Knowles) placed near the left and right wings, respectively. Microphone signals were amplified with a custom amplifier^54^ and recorded at 10 kHz. Wing movement was monitored with a camera (cat. no. BFS-U3-04S2M-CS, Teledyne FLIR) equipped with a periscopic lens (InfiniStix 90°, working distance 94 mm, magnification ×1.0, Infinity). Flies were illuminated by infrared light (850 nm) (cat. no. M850F2, Thorlabs) with a patch cable (cat. no. M125L01, Thorlabs). A long-pass (cat. no. FEL0800, Thorlabs) and a short-pass (cat. no. FES0850, Thorlabs) filter were placed on the lens to remove the optogenetic and two-photon activation light, respectively. Video was recorded at 100 frames per s.

In the experiment where CsChrimson and jGCaMP7f were expressed in the same neurons (Extended Data Fig. 3n,o), tetrodotoxin was added to the saline (final concentration of 1 μM; Tetrodotoxin citrate, Tocris Bioscience) to eliminate the effects of chemical synaptic transmission from other neurons. To promote the delivery of tetrodotoxin to neurons in the VNC, we cut a prothoracic leg nerve with forceps.

In the wing cut experiment (Extended Data Fig. 4k–m), we first recorded calcium signals in the flies with intact wings. We then transferred the fly to a dissection microscope, cut both wings near the wing hinges with forceps and recorded calcium signals again. The flies used in the control experiment underwent the same procedure except that the wings were not cut.

### Immunohistochemistry

The dissections, immunohistochemistry and imaging of fly central nervous systems were performed using protocols described previously^49,56^ (https://www.janelia.org/project-team/flylight/protocols) with modifications. Briefly, brains and VNCs were dissected in Schneider’s insect medium and fixed in 2% paraformaldehyde at room temperature for 55 min. Tissues were washed in PBT (0.5% Triton X-100 in PBS) and blocked using 5% normal goat serum before incubation with antibodies.

To visualize the expression pattern of GFP or GCaMP, tissues were stained using rabbit anti-GFP (1:1,000, cat. no. A-11122, Thermo Fisher Scientific) and mouse nc82 (1:30, Developmental Studies Hybridoma Bank) as primary antibodies and Alexa Fluor 488-conjugated goat anti-rabbit (1:800, cat. no. A-11034, Thermo Fisher Scientific) and Alexa Fluor 568-conjugated goat anti-mouse (1:400, cat. no. A-11031, Thermo Fisher Scientific) as secondary antibodies. To visualize the expression pattern of tdTomato, tissues were stained using rabbit anti-dsRed (1:1,000, cat. no. 632496, Clontech) and mouse nc82 (1:30) as primary antibodies and Cy3-conjugated goat anti-rabbit (1:1,000, cat. no. 111-165-144, Jackson ImmunoResearch) and Cy2-conjugated goat anti-mouse (1:600, cat. no. 115-225-166, Jackson ImmunoResearch) as secondary antibodies. To visualize the expression pattern of GCaMP and tdTomato in the same samples, tissues were stained using chicken anti-GFP (1:1,000, cat. no. A-10262, Thermo Fisher Scientific), rabbit anti-dsRed (1:1,000) and mouse nc82 (1:30) as primary antibodies and Alexa Fluor 488-conjugated goat anti-chicken (1:800, cat. no. A32931, Thermo Fisher Scientific), Cy3-conjugated goat anti-rabbit and Cy5-conjugated goat anti-mouse (1:500, cat. no. 115-175-166, Jackson ImmunoResearch) as secondary antibodies. To visualize the expression pattern of *dsx*, tissues were stained using mouse anti-DsxDBD (1:2, Developmental Studies Hybridoma Bank), rabbit anti-GFP (1:1,000) and rat anti-DN-Cadherin (1:100, DN-Ex #8, Developmental Studies Hybridoma Bank) as primary antibodies and Cy3-conjugated goat anti-mouse (1:800, cat. no. 115-165-166, Jackson ImmunoResearch), Alexa Fluor 488-conjugated goat anti-rabbit and Cy5-conjugated goat anti-rat (1:500, cat. no. 112-175-167, Jackson ImmunoResearch) as secondary antibodies. Flies for multi-color flip out^49^ underwent a 40-min heat shock during 0–1 d post eclosion and were dissected at 5–14 d old. These tissues were stained using the protocol described in ref. ^49^, available at https://www.janelia.org/project-team/flylight/protocols (Protocol: 'IHC - MCFO').

Image stacks were collected using a confocal microscope (LSM710, Zeiss) with an objective lens (Plan-Apochromat ×20/0.8 M27 or Plan-Apochromat ×40/1.3 M27, Zeiss). ImageJ2 (v.2.9.0) was used to analyze the images.

### Song-type classification

Pulse and sine songs were detected using SongExplorer^57^, a deep-learning-based algorithm for segmenting acoustic communication signals. We first removed low-frequency components of the microphone signals (<100 Hz), which were mostly irrelevant for song, using continuous wavelet transformation. We then performed manual annotation for a subset of the audio data. For the data obtained from freely moving flies, we used the labels ‘pulse’, ‘sine’, ‘inter-pulse intervals’, ‘others’ for nonsong sounds (for example, noise during grooming) and ‘ambient’. Another label, ‘flight’, was added for the data from tethered flies because these flies occasionally showed flight-like wing movement. We then trained a classifier separately for freely moving, tethered flies during the recording from the VNC, and tethered flies during the recording from the brain, using the annotated data. These classifiers were applied to assign a label at every 1.6 ms of the data from all the recordings. The classification accuracies, measured with cross-validation, for the data from freely moving and tethered flies were 91.6% and 88.1%, respectively. Visual inspection of the classifier predictions revealed that misclassifications often occurred at a smaller number of consecutive time bins compared with correct predictions. To correct for these misclassifications, we smoothed the time series of classifier predictions with median filters (the window size for detecting ‘pulse’, 17.6 ms; ‘sine’, 25.6 ms; ‘flight’, 80 ms).

To compare the amounts of pulse and sine song, we calculated the pulse train based on the classifier predictions. Pulse song is composed of discrete pulse events separated by inter-pulse intervals, whereas sine song is continuous. We defined the pulse train by merging pulse events with inter-pulse intervals of 50 ms or shorter. This allows comparisons of the amounts of pulse and sine song on the same scale. Events designated as ‘pulse’ in the figures represent the pulse train.

### Data analysis for behavioral experiments

For each recording, we calculated the time series of the proportions of pulse and sine song by combining data across repeated presentations of the same optogenetic stimulus. These time series were averaged across flies to obtain the time course of song proportions, or across time to calculate the song probabilities for each fly. For the paired fly optogenetic activation experiment, we quantified how much optogenetic stimulation changed the amounts of pulse and sine song by subtracting the proportions of pulse and sine song during optogenetic stimulation from those during 5-s pre-stimulation periods (Extended Data Fig. 9b,c). We also quantified the probabilities of song initiation, song termination and song-type switch based on the song-type classifier’s output (Fig. 7d,e and Extended Data Fig. 9d–g). For the analysis of song initiation, we detected the ‘ambient’ events and calculated the probabilities of observing ‘pulse’ and ‘sine’ events in the following time bin. The probabilities of pulse song termination were calculated by detecting the ‘pulse’ events and calculating the probabilities of observing ‘ambient’ events in the following time bin. The probabilities of pulse-to-sine transitions were computed using the same method except that ‘ambient’ events were replaced with ‘sine’ events. The probabilities of sine song termination and sine-to-pulse transitions were calculated similarly. To construct the time courses of these probabilities (Fig. 7d,e and Extended Data Fig. 9d), we separated data into 150-ms bins, aggregated data across flies and calculated the probabilities for each bin. To quantify the optogenetic stimulation effects on these probabilities on a fly-by-fly basis (Extended Data Fig. 9e–g), we subtracted the probabilities calculated based on the data during optogenetic stimulation from those during 5-s pre-stimulation periods.

### Data analysis for calcium imaging experiments

Time series of *z*-stack images underwent rigid motion correction with the NoRMCorre algorithm^58^. To analyze calcium signals of dPR1 cell bodies, we first set a volume of interest (VOI) separately for the dPR1 neuron in each hemisphere. We then calculated an average intensity *z*-projection of the VOI and performed another round of rigid motion correction. The same procedure was carried out for analyzing TN1A-2 cell bodies, dPR1 neurite, TN1A-2 neurite, pIP10 neurite, pMP2 neurite and vPR9 neurite, except that a single VOI was used to calculate an average intensity *z*-projection. For each recording, regions of interest (ROIs), each corresponding to a cell body or target neurites, were manually defined using an average image of the motion-corrected *z*-projections. Each ROI for pIP10 neurites contained signals from the pIP10 neurons in both hemispheres. Each ROI for pMP2 neurites reflected signals from one pMP2 neuron. Signals in each ROI were averaged for further analysis. Motion corrections were performed using either GCaMP or tdTomato signals. For analyzing data from pan-neuronal and *fru*-expressing neuron imaging, the motion-corrected *z*-stack images went through 2-by-2 voxel binning in the *xy*-planes to increase the signal-to-noise ratio. Δ*F*/*F*, the change in fluorescence intensity divided by the mean fluorescent signal during the 10-s period preceding optogenetic stimulation, was calculated for each ROI or voxel.

To compare spatial activity patterns across flies, inter-fly image registration was performed for the data from pan-neuronal and *fru*-expressing neuron imaging. First, the motion-corrected *z*-stack images were averaged across time for each recording. Second, the averaged *z*-stack images went through 2-by-2 voxel binning in the *xy*-planes. Third, the binned images from one recording were selected as a reference for each target volume (for example, a dorsal volume) and each driver line (for example, the pan-neuronal line). Finally, the time-averaged *z*-stack images of each recording were aligned to the reference with either the NoRMCorre rigid registration or the Computational Morphometry Toolkit^59^ run with a Fiji plugin (https://github.com/jefferis/fiji-cmtk-gui). These registration parameters were used to calculate population-averaged calcium response patterns.

To analyze the relationship between calcium signals and song, we converted the sampling rate of song classifier predictions (625 Hz) to that of calcium imaging (7.1 Hz; sampling at every 141 ms) as follows. Time series of the predictions were separated into 141-ms bins, and the mean probabilities of each event (‘pulse’, ‘sine’, ‘ambient’ and ‘flight’) were calculated for each bin. We considered that an event occurred in a bin if the probability exceeded a threshold (0.1 for ‘pulse’, 0.6 for ‘sine’, 0.9 for ‘ambient’, 0.01 for ‘flight’). Defining event occurrence with these thresholds rarely gave false positives when assessed with visual inspection. We excluded the data during ‘flight’ and from −1 to 1 s after ‘flight’ in the analysis of song-type transitions.

To characterize the changes in calcium signals during song-type transitions, we first calculated the average Δ*F*/*F* in the two bins over which song type was changed (from 141 ms before the transition to 141 ms after the transition). We then subtracted this value from the average Δ*F*/*F* in the third and fourth bins after the change in song type (282–564 ms after the transition) for the analysis of cell body signals. To take into account faster GCaMP kinetics at neurites than cell bodies^60^, the average Δ*F*/*F* in the second and third bins after the change in song type (141–423 ms after the transition) was used for the subtraction in the analysis of neurite signals. Considering the kinetics of jGCaMP7f (ref. ^37^), this quantity likely reflects changes in neural activity during song-type transitions. We averaged this quantity across transition events and divided it by the average Δ*F*/*F* in the two bins over which song type was changed, obtaining the normalized mean change in Δ*F*/*F* during song-type transitions for each ROI. To characterize the changes in calcium signals during quiet-to-pulse transitions, we detected ‘pulse’ bins that followed at least 2-s continuous ‘ambient’ periods. The changes in Δ*F*/*F* during the transitions were defined as the subtraction of the average Δ*F*/*F* during the 2-s period before the transition from that after the transition. We analyzed the data from the recordings where the transitions occurred at least five times for the analysis of song-type transitions and two times for the analysis of quiet-to-pulse transitions. We focused on song-type transitions, rather than overall calcium signals during each song type, to avoid false detection of song-type selectivity due to slow GCaMP kinetics. Constant neural activity can lead to growing GCaMP signals over several seconds due to temporal summation^37^. This signal growth could generate differential levels of calcium signals between song types because pulse and sine song tended to occur in different timings in 10-s stimulation periods (for example, Extended Data Fig. 3a,h). To examine the relationship between transition-related activity and song bout lengths (Extended Data Fig. 3f,m), we separated data based on the bout lengths before and after the transitions (short and long pulse bouts, pulse bouts shorter and longer than 422 ms, respectively; short and long sine bouts, sine bouts shorter and longer than 704 ms, respectively).

To compare the decay kinetics of dPR1’s GCaMP signals during sine song and after the song driver stimulation offset (Extended Data Fig. 3g), we first calculated the average time course of GCaMP signals during the transitions from pulse song to long bouts of sine song. We then computed the half decay time of the GCaMP signals by fitting a one-term exponential function to the data during sine song (845 ms, 6 bins; pink shaded region in Extended Data Fig. 3g). To compute the half decay time after the optogenetic stimulation offset, we first calculated the time course of calcium signals averaged across trials and stimulation levels for each fly. We then averaged the time courses across flies and fitted a one-term exponential function to the data during a period where flies stopped singing almost completely (845 ms, 6 bins; pink shaded region in Extended Data Fig. 3g).

To assess whether individual TN1 neurons responded to optogenetic stimulation, we calculated the mean Δ*F*/*F* during the stimulation period subtracted from that of the 10-s pre-stimulation period for each trial for a range of stimulation levels (9.8–17.1 μW mm^−2^ for song driver stimulation; 19.5–156.2 μW mm^−2^ for pIP10 stimulation). We then aggregated the responses across stimulation levels and performed a two-sided one-sample *t*-test. The same test was performed for dPR1 and TN1A-2 neurons and found that all of these neurons displayed significant responses (*P* < 0.05). We also tested pIP10 and pMP2 with a range of stimulation levels (230.2–575.6 μW mm^−2^) and found that all but two pMP2 neurons showed significant responses (*P* < 0.05). These two pMP2 neurons were excluded from further analysis.

For single dPR1, TN1A-2 and TN1 neurons, the preference for song type was quantified using the receiver operating characteristic (ROC) analysis (Extended Data Fig. 5a). We first built the distribution of the changes in Δ*F*/*F* separately for pulse-to-sine and sine-to-pulse transitions. We then used the two distributions to calculate the area under the ROC curve. An index termed the song-type preference was defined as the area under the curve scaled from −1 to 1. This index quantifies how much the type of transition (pulse-to-sine versus sine-to-pulse) can be predicted based on the changes in calcium signals. Song-type preferences of 1 and −1 represent perfect predictions with higher calcium signals for pulse and sine song, respectively. A song-type preference of 0 indicates that the changes in calcium signals were indistinguishable between the two types of transitions. For single TN1A-2 neurons, we assessed whether the song-type preference was different from 0 using a permutation test. We shuffled the labels (that is, pulse-to-sine and sine-to-pulse transitions) of transition responses and calculated song-type preference. This was repeated 10,000 times to obtain a null distribution of song-type preference. The same permutation test was conducted for each voxel for the data obtained in pan-neuronal and *fru*-expressing neuron imaging (Figs. 4 and 5). For these data, we also calculated a null distribution of transition responses for each voxel using permutation (Figs. 4 and 5). We first randomized the relationship between song-type transitions and calcium signals by shuffling the trials for calcium signal data. We then calculated the distribution of transition responses as in the original data.

Images involved in calcium signals were spatially smoothed with a two-dimensional Gaussian filter for presentation in figures.

### Connectomic and morphological analysis

All connectomic and neuron skeleton data were obtained from the male adult nerve cord dataset^27,28,29^ using neuPrint^61^. Only the neurons that had been traced and possessed at least 100 presynapses or postsynapses were analyzed. Electron microscopy-reconstructed images of single neurons were created by registering neuron skeletons to a VNC template made from light microcopy images^62^. To create the *t*-distributed stochastic neighbor embedding (*t*-SNE) plot from the connectivity matrix among pIP10, pMP2, dPR1 and TN1A-2 neurons (Extended Data Fig. 10b), we took each neuron and constructed a vector that represents the number of input synapses to this neuron from each neuron and the number of output synapses from this neuron to each of the other neurons. We then performed *t*-SNE using the cosine distance metric with the perplexity of 10. A list of body IDs for the neurons analyzed is provided in Supplementary Data 1. To construct the plot of predicted neurotransmitters (Extended Data Fig. 10a), we obtained the probability of each neurotransmitter type for each synapse using neuPrint^61^. We then averaged these probabilities across synapses for each neuron.

### Statistics

All statistical tests were two-tailed and performed using MATLAB 2022b. Sample size was determined based on effect sizes and sample-by-sample variability observed in pilot experiments. Data distribution was assumed to be normal but this was not formally tested. Flies were randomly chosen from culture vials and were allocated to experimental groups based on genotypes. Different levels of optogenetic stimulation were delivered in a random order during calcium imaging. Such randomization was not performed during behavioral experiments because the effects of optogenetic stimulation were acute and not history-dependent in pilot experiments. Data collection and analysis were not performed blind to the conditions of the experiments. Blinding was not practical, and the same, automated data collection and analysis pipelines were used across different experimental conditions.

### Reporting summary

Further information on research design is available in the Nature Portfolio Reporting Summary linked to this article.

## Online content

Any methods, additional references, Nature Portfolio reporting summaries, source data, extended data, supplementary information, acknowledgements, peer review information; details of author contributions and competing interests; and statements of data and code availability are available at 10.1038/s41593-024-01738-9.

## Supplementary information

Reporting Summary

Supplementary Video 1 An example trial of calcium imaging in the neurite of dPR1 neurons. Top left, a bottom view of the fly. The left side of the image corresponds to the right side of the fly. Bottom left, the microphone signal for the right wing. Top right, Δ*F*/*F* averaged across *z*-planes. The left side of the image corresponds to the left side of the VNC. Top left, schematic of the imaged volume. Red boxes represent the timing of optogenetic stimulation.

Supplementary Video 2 An example trial of calcium imaging in the neurite of TN1A-2 neurons. Same format as in Supplementary Video 1.

Supplementary Video 3 An example trial of pan-neuronal calcium imaging in dPR1 neurons. Top left, the microphone signal for the right wing. The red box represents the timing of optogenetic stimulation. Bottom left, schematic of the imaged volume. Right, raw fluorescent signals (*F*) for each *z*-plane.

Supplementary Video 4 Same as in Supplementary Video 3 but for calcium signals averaged across *z*-planes.

Supplementary Data A list of body IDs for pIP10, pMP2, dPR1, TN1A-1 and TN1A-2 neurons in the MANC connectome dataset.

## Data Availability

Data generated in this study are available in Figshare with the identifier 10.25378/janelia.25041485.v1 (ref. ^63^). The connectomic data^27,28,29^ are available at https://neuprint.janelia.org.

## Code availability

Code is available via Figshare at 10.25378/janelia.25041485.v1 (ref. ^63^) and Zenodo at 10.5281/zenodo.11493396 (ref. ^64^).
