# Supplementary material for: Activity of nested neural circuits drives different courtship songs in Drosophila
Source: Nat Neurosci. 2024 Aug 28;27(10):1954–65. doi: 10.1038/s41593-024-01738-9 (PMC11452343; doi:10.1038/s41593-024-01738-9)
Supplement: Supplementary file 1 — Reporting Summary [file 41593_2024_1738_MOESM1_ESM.pdf]

nature portfolio

Corresponding author(s): Hiroshi Shiozaki, David Stern

Last updated by author(s): Jun 5, 2024

Reporting Summary

Nature Portfolio wishes to improve the reproducibility of the work that we publish. This form provides structure for consistency and transparency in reporting. For further information on Nature Portfolio policies, see our Editorial Policies and the Editorial Policy Checklist.

Statistics

For all statistical analyses, confirm that the following items are present in the figure legend, table legend, main text, or Methods section.

n/a	Confirmed	
☐	☒	The exact sample size (*n*) for each experimental group/condition, given as a discrete number and unit of measurement
☐	☒	A statement on whether measurements were taken from distinct samples or whether the same sample was measured repeatedly
☐	☒	The statistical test(s) used AND whether they are one- or two-sided<br>*Only common tests should be described solely by name; describe more complex techniques in the Methods section.*
☒	☐	A description of all covariates tested
☐	☒	A description of any assumptions or corrections, such as tests of normality and adjustment for multiple comparisons
☐	☒	A full description of the statistical parameters including central tendency (e.g. means) or other basic estimates (e.g. regression coefficient) AND variation (e.g. standard deviation) or associated estimates of uncertainty (e.g. confidence intervals)
☐	☒	For null hypothesis testing, the test statistic (e.g. *F*, *t*, *r*) with confidence intervals, effect sizes, degrees of freedom and *P* value noted<br>*Give P values as exact values whenever suitable.*
☒	☐	For Bayesian analysis, information on the choice of priors and Markov chain Monte Carlo settings
☒	☐	For hierarchical and complex designs, identification of the appropriate level for tests and full reporting of outcomes
☐	☒	Estimates of effect sizes (e.g. Cohen's *d*, Pearson's *r*), indicating how they were calculated

Our web collection on statistics for biologists contains articles on many of the points above.

Software and code

Policy information about availability of computer code

Data collection	MATLAB (2016b, 2019a) and ScanImage (2019a) were used to collect two-photon imaging data. MATLAB (2016b) was used to collect behavioral data.
Data analysis	MATLAB (2022a), NoRMCorre, and ImageJ2 (ver. 2.9.0) were used to analyze data. Code used in data analysis is available at Figshare (http://doi.org/10.25378/janelia.25041485) and Zenodo (https://doi.org/10.5281/zenodo.11493396).

For manuscripts utilizing custom algorithms or software that are central to the research but not yet described in published literature, software must be made available to editors and reviewers. We strongly encourage code deposition in a community repository (e.g. GitHub). See the Nature Portfolio guidelines for submitting code & software for further information.

Data

Policy information about availability of data

All manuscripts must include a data availability statement. This statement should provide the following information, where applicable:

- Accession codes, unique identifiers, or web links for publicly available datasets
- A description of any restrictions on data availability
- For clinical datasets or third party data, please ensure that the statement adheres to our policy

Data generated in this study are available in Figshare with the identifier https://doi.org/10.25378/janelia.25041485.v1. The connectomic data are available at https://neuprint.janelia.org.

Research involving human participants, their data, or biological material

Policy information about studies with human participants or human data. See also policy information about sex, gender (identity/presentation), and sexual orientation and race, ethnicity and racism.

Reporting on sex and gender	N/A
Reporting on race, ethnicity, or other socially relevant groupings	N/A
Population characteristics	N/A
Recruitment	N/A
Ethics oversight	N/A

Note that full information on the approval of the study protocol must also be provided in the manuscript.

Field-specific reporting

Please select the one below that is the best fit for your research. If you are not sure, read the appropriate sections before making your selection.

☒ Life sciences ☐ Behavioural & social sciences ☐ Ecological, evolutionary & environmental sciences

For a reference copy of the document with all sections, see nature.com/documents/nr-reporting-summary-flat.pdf

Life sciences study design

All studies must disclose on these points even when the disclosure is negative.

Sample size	Sample size was determined based on effect sizes and sample-by-sample variability observed in pilot experiments.
Data exclusions	We excluded flies from data analysis when these flies failed to produce sufficient amounts of pulse and/or sine song, or data when flies exhibited flight behavior as described in Methods.
Replication	Each experimental finding was replicated in multiple flies as shown in figures. Only representative data were shown for immunostaining experiments because the high reproducibility of the method is widely recognized in the field.
Randomization	Flies were randomly chosen from culture vials and were allocated experimental groups based on genotypes. Different levels of optogenetic stimulation were delivered in a random order during calcium imaging. Such randomization was not performed during behavioral experiments because the effects of optogenetic stimulation were acute and not history-dependent in pilot experiments.
Blinding	Data collection and analysis were not performed blind to the conditions of the experiments. Blinding was not practical, and the same, automated data collection and analysis pipelines were used across different experimental conditions.

Reporting for specific materials, systems and methods

We require information from authors about some types of materials, experimental systems and methods used in many studies. Here, indicate whether each material, system or method listed is relevant to your study. If you are not sure if a list item applies to your research, read the appropriate section before selecting a response.

Materials & experimental systems

n/a	Involved in the study	
☐	☒	Antibodies
☒	☐	Eukaryotic cell lines
☒	☐	Palaeontology and archaeology
☐	☒	Animals and other organisms
☒	☐	Clinical data
☒	☐	Dual use research of concern
☒	☐	Plants

Methods

n/a	Involved in the study	
☒	☐	ChIP-seq
☒	☐	Flow cytometry
☒	☐	MRI-based neuroimaging

Antibodies

Antibodies used	rabbit anti-GFP (1:1000, A-11122, Thermo Fisher Scientific), mouse nc82 (1:30, Cat# nc82, Developmental Studies Hybridoma Bank), Alexa Fluor 488-<br>conjugated goat anti-rabbit (A-11034, Thermo Fisher Scientific), Alexa Fluor 568-conjugated goat anti-mouse (A-11031, Thermo Fisher Scientific), rabbit anti-dsRed (#632496, Clontech), Cy3-conjugated goat anti-rabbit (#111-165-144, Jackson ImmunoResearch), Cy2-conjugated goat anti-mouse (#115-225-166, Jackson ImmunoResearch), chicken anti-GFP (A-10262, Thermo Fisher Scientific), Alexa Fluor 488-conjugated goat anti-chicken (A32931, Thermo Fisher Scientific), Cy5-conjugated goat anti-mouse (#115-175-166, Jackson ImmunoResearch), mouse anti-DsxDBD (Developmental Studies Hybridoma Bank), rat anti-DN-Cadherin (DN-Ex #8, Developmental Studies Hybridoma Bank), Cy3-conjugated goat anti-mouse (#115-165-166, Jackson ImmunoResearch), Cy5-conjugated goat anti-rat (#112-175-167, Jackson ImmunoResearch)
Validation	All the primary antibodies used in this study have been applied to the Drosophila nervous system in previous studies. Validation information can be found at manufacturers' websites at https://www.thermofisher.com/antibody/product/GFP-Antibody-Polyclonal/A-11122 (rabbit anti-GFP), https://dshb.biology.uiowa.edu/nc82 (mouse nc82), https://www.takarabio.com/products/antibodies-and-elisa/fluorescent-protein-antibodies/red-fluorescent-protein-antibodies (rabbit anti-dsRed), https://www.thermofisher.com/antibody/product/GFP-Antibody-Polyclonal/A10262 (chicken anti-GFP), https://dshb.biology.uiowa.edu/DsxDBD (mouse anti-DsxDBD), https://dshb.biology.uiowa.edu/DN-Ex-8 (rat anti-DN-Cadherin).

Animals and other research organisms

Policy information about studies involving animals; ARRIVE guidelines recommended for reporting animal research, and Sex and Gender in Research

Laboratory animals	Drosophila melanogaster 5–7 days after eclosion. Details of the genotypes are described in Method.
Wild animals	No wild animals were used in the study.
Reporting on sex	Findings apply only to males as we studied male-specific behavior.
Field-collected samples	No field collected samples were used in the study.
Ethics oversight	No ethical approval or guidance was required because we used Drosophila.

Note that full information on the approval of the study protocol must also be provided in the manuscript.

Plants

Seed stocks	N/A
Novel plant genotypes	N/A
Authentication	N/A
